# Assessment of multi-population polygenic risk scores for lipid traits in African Americans

**DOI:** 10.7717/peerj.14910

**Published:** 2023-05-16

**Authors:** Domenica E. Drouet, Shiying Liu, Dana C. Crawford

**Affiliations:** 1 Department of Medicine, Case Western Reserve University, Cleveland, OH, United States of America; 2 Population and Quantitative Health Sciences, Case Western Reserve University, Cleveland, OH, United States of America; 3 Cleveland Institute for Computational Biology, Case Western Reserve University, Cleveland, OH, United States of America; 4 Genetics and Genome Sciences, Case Western Reserve University, Cleveland, OH, United States of America

**Keywords:** Lipids, Polygenic risk scores, African Americans, Genetic risk scores, Electronic health records, Biorepository, Metabochip

## Abstract

Polygenic risk scores (PRS) based on genome-wide discoveries are promising predictors or classifiers of disease development, severity, and/or progression for common clinical outcomes. A major limitation of most risk scores is the paucity of genome-wide discoveries in diverse populations, prompting an emphasis to generate these needed data for trans-population and population-specific PRS construction. Given diverse genome-wide discoveries are just now being completed, there has been little opportunity for PRS to be evaluated in diverse populations independent from the discovery efforts. To fill this gap, we leverage here summary data from a recent genome-wide discovery study of lipid traits (HDL-C, LDL-C, triglycerides, and total cholesterol) conducted in diverse populations represented by African Americans, Hispanics, Asians, Native Hawaiians, Native Americans, and others by the Population Architecture using Genomics and Epidemiology (PAGE) Study. We constructed lipid trait PRS using PAGE Study published genetic variants and weights in an independent African American adult patient population linked to de-identified electronic health records and genotypes from the Illumina Metabochip (*n* = 3,254). Using multi-population lipid trait PRS, we assessed levels of association for their respective lipid traits, clinical outcomes (cardiovascular disease and type 2 diabetes), and common clinical labs. While none of the multi-population PRS were strongly associated with the tested trait or outcome, PRS_LDL-C_was nominally associated with cardiovascular disease. These data demonstrate the complexity in applying PRS to real-world clinical data even when data from multiple populations are available.

## Introduction

Polygenic risk scores (PRS) are generally defined as the sum of an individual’s additive genetic risk for an outcome or disease of interest (Cooke Bailey & Igo Jr, 2016; Igo Jr, Kinzy & Cooke Bailey, 2019; Osterman, Kinzy & Cooke Bailey, 2021). Early genetic risk scores for cardiovascular disease (Kathiresan et al., 2008), type 2 diabetes (Hivert et al., 2011), and age-related macular degeneration (Osterman, Kinzy & Cooke Bailey, 2021), to name a few, suggest genome-wide association study (GWAS)-identified variants in aggregate have the potential for patient risk reclassification or identification prior to development of disease or progression to severe disease, spurring interest in the development of clinical applications of these basic research findings (Torkamani, Wineiger & Topol, 2018; Christoffersen & Tybjærg-Hansen, 2021). Despite the promising preliminary data and the now 15 years’ worth of genome-wide discoveries, PRS are still largely limited to or based on GWAS data from European-descent populations. Recent initiatives that encourage genomic discovery in diverse populations will enable the assessment of multi-population PRS for outcomes or traits as population-specific and shared genetic variants emerge from the most recent GWAS stemming from these efforts (Hindorff et al., 2018).

The Population Architecture using Genomics and Epidemiology (PAGE) Study, supported by the National Human Genome Research Institute (NHGRI), was first established to generalize GWAS findings in diverse populations (Dumitrescu et al., 2011; Matise et al., 2011; Carlson et al., 2013). The PAGE Study has since expanded to include discovery of genetic associations in diverse populations for common outcomes and traits (Wojcik et al., 2019). In one such recent PAGE Study, array-based GWAS for high density lipoprotein cholesterol (HDL-C), low-density lipoprotein cholesterol (LDL-C), triglycerides (TG), and total cholesterol (TC), common clinical biomarkers ordered to assess risk for the development of a variety of outcomes including cardiovascular disease (CVD) and type 2 diabetes (T2D), was conducted in > 45,000 ancestrally diverse participants (Hu et al., 2020). A total of nine novel lipid loci were identified. Further, comparisons of effect sizes across populations revealed smaller genetic effects in African Americans and Hispanic compared with the same genetic associations identified in European descent participants (Hu et al., 2020).

The genetic associations identified for lipid traits in the PAGE Study provide a still unique opportunity to calculate and apply multi-population lipid trait PRS for diverse populations. To seize this opportunity, we accessed clinical data from electronic health records (EHRs) linked to a biorepository with genetic data for almost 12,000 African American patients (Crawford et al., 2015). Here, we calculate multi-population PRS for lipids based on the PAGE Study discovery efforts and apply them to real world clinical data in an independent African American patient sample. We anticipated that the availability of genetic association data from diverse populations that include African Americans would result in robust PRS. The resulting data highlight many of the challenges that need to be addressed before PRS research can be translated into clinical practice (Li et al., 2020).

## Materials & Methods

### Study population

We accessed an existing and previously described dataset (Crawford et al., 2015) of de-identified EHRs linked to biospecimens genotyped on the Illumina Metabochip (Buyske et al., 2012; Voight et al., 2012). These data are a subset of the larger BioVU, Vanderbilt University Medical Center’s (VUMC) biobank established in 2007 (Roden et al., 2008).

BioVU links discarded biospecimens collected for clinical purposes during outpatient visits to a de-identified version of the patient’s EHR. The de-identification process includes the scrubbing of identifiers, data shifting, and linkage to biospecimens *via* a one-way hash that combined make re-identification difficult and unlikely (Roden et al., 2008). BioVU as a VUMC program has received approval from the Vanderbilt Institutional Review Board (IRB) and was reviewed in detail by the federal Office for Human Research Protections (OHRP), who agreed with the non-human subjects regulatory designation for both the resource and subsequent research (Roden et al., 2008).

The data accessed for the current study were collected under an opt-out participation model described to the patient annually as part of the consent to treat process and represent DNA samples extracted from blood collected between 2007 and 2011 and clinical data collected through 2011. As a study site of the PAGE Study, a consortium focused on genetic association studies in diverse populations (Matise et al., 2011), we selected at the time all DNA samples in BioVU from non-European descent patients for Illumina Metabochip genotyping (*n* = 15,863), the majority of which are from African American or Black patients (73%; Crawford et al., 2015). As we have previously described (Crawford et al., 2015), BioVU is representative of the Nashville, Tennessee VUMC patient population, a population that is on average older and of European descent compared with the surrounding Davidson County population. The dataset described here is de-identified and is considered to be non-human subjects research (Vanderbilt University IRB #110579) (Pulley et al., 2010).

### Electronic phenotyping

Analyses were limited to African American adults aged 18 years or older. All demographic and clinical data were extracted using a combination of structured (*e.g.*, International Classification of Diseases 9th edition, Clinical Modification (ICD-9-CM) codes, Current Procedural Terminology (CPT) codes, problems lists, and laboratory values) and unstructured (clinical notes) data available in the EHR.  Race/ethnicity in BioVU was administratively assigned, and these assignments are highly correlated with genetic ancestry for some (*e.g.*, European and African Americans) but not all (*e.g.*, Hispanics and East Asians) groups (Dumitrescu et al., 2010; Hall et al., 2014; Farber-Eger et al., 2017). Age was calculated as the age of the patient in 2011 based on the patient’s year of birth. First mention (*e.g.*, the first clinic visit associated with a lipid lab result) of each patient’s serum HDL-C, LDL-C, TG, and TC was extracted and recorded as either a “pre-medication” or “post-medication” value. Pre-medication values were defined as free of evidence of concurrent lipid lowering medication usage. As previously described (Crawford et al., 2015), lipid lowering medications included statins (also known as HMG CoA reductase inhibitors, atorvastatin (Lipitor^®^), fluvastatin (Lescol^®^), lovastatin (Mevacor^®^, Altoprev™), pravastatin (Pravachol^®^), rosuvastatin calcium (Crestor^®^), simvastatin (Zocor^®^), lovastatin + niacin (Advicor^®^), atorvastatin + amlodipine (Caduet^®^), and simvastatin + ezetimibe (Vytorin™)), selective cholesterol absorption inhibitors (ezetimibe (Zetia^®^)), resins (cholestyramine (Questran^®^, Questran^®^ Light, Prevalite^®^, Locholest^®^, Locholest^®^ Light), colestipol (Colestid^®^), colesevelam Hcl (WelChol^®^)), fibrates (gemfibrozil (Lopid^®^), fenofibrate (Antara^®^, Lofibra^®^, Tricor^®^, and Triglide™), clofibrate (Atromid-S)), and niacin. Fasting status is unknown. Heights and weights closest to the first mention of the lipid trait were extracted to calculate body mass index (BMI; kg/m^2^). Prevalent cardiovascular disease was defined as mention of heart attack keywords in the problems list (“MI”, “myocardial infarction”, or “heart attack”), CPT codes (Crawford et al., 2018) for coronary artery bypass graft (33510–33514; 33515; 33516; 33517–33519; 33520; 33521–33523; and 33534–33536) and angioplasty and/or coronary stents (92980–92981; 92982; 92984; 92995; and 92996), or three mentions of International Classification of Diseases (ICD)-9-CM codes (410 or 410.*) (Dumitrescu et al., 2015a) for myocardial infarction. Type 2 diabetes cases status was defined using a combination of ICD codes, labs, and medications from the previously described and validated electronic Medical Records & Genomics (eMERGE) Network algorithm (Kho et al., 2012).

### Genotyping and quality control

The Illumina Metabochip was genotyped by Vanderbilt Technologies for Advanced Genomics (VANTAGE), formerly the Vanderbilt University Center for Human Genetics Research DNA Resources Core, for the Epidemiologic Architecture for Genes Linked to Environment (EAGLE) study, a study site of PAGE I (Matise et al., 2011). Genotyping was conducted following the manufacturer’s protocol (Illumina, Inc.; San Diego, CA.). Per PAGE I protocol, 360 HapMap samples were also genotyped for quality control (Crawford et al., 2012). Genotype calling and standard quality control measures (call rates, duplicate checks, Hardy-Weinberg Equilibrium, *etc.*) have been described by Buyske et al. 2011 and more recently by Kaur et al. (2021). Metabochip genotypes linked to the EAGLE BioVU de-identified clinical data are available through the database of Genotypes and Phenotypes (dbGaP) accession number phs002767.v1.p1.

### Polygenic risk score calculations

Unweighted and weighted multi-population PRS for HDL-C, LDL-C, TG, and TC were calculated for each patient based on published summary statistics for diverse populations from the PAGE Study (Hu et al., 2020). We first extracted Metabochip genotypes available for SNPs previously associated HDL-C (44), LDL-C (36), TG (51), TC (48) reported by the PAGE Study at genome-wide significance (*p* ≤ 5  ×  10^−8^) (Table S7 from Hu et al., 2020; Supplementary Table). After removing SNPs out of Hardy Weinberg Equilibrium at *p* < 10^−4^, we calculated unweighted PRS for each patient and each lipid trait by counting the number of risk alleles (N) for 42, 34, 50, and 46 HDL-C, LDL-C, TG, and TC-associated SNPs (k), respectively. PRS were weighted (PRS_w_) using the absolute value of the betas (|*β*|) for the corresponding associations published by the PAGE Study (Hu et al., 2020).

\begin{eqnarray*}{\text{PRS}}_{\text{w}}=\sum _{i=1}^{k}{|}{\beta }_{i}{|}{N}_{i}. \end{eqnarray*}

### Statistical methods

Principal components (PCs) were generated as previously described (Buyske et al., 2012; Kaur et al., 2021). We tested each SNP for an association with the lipid trait previously associated as described in Hu et al. (2020). Single SNP tests of association were performed in PLINK version 1.90 using linear regression, unadjusted and adjusted for sex, age, BMI, and the first ten PCs. TG levels were transformed (natural log) prior to tests of association. Summary statistics were visualized using Synthesis View (Pendergrass et al., 2010b; Pendergrass et al., 2010a; Supplementary File). We also tested for associations between the lipid traits and their respective unweighted and weighted PRS using linear regression, unadjusted and adjusted for age, sex, BMI, and the first ten PCs. Tests of association between CVD and T2D and unweighted and weighted PRS were performed using 2x2 tables (unadjusted) and logistic regression (CVD and LDL-C weighted PRS adjusted by LDL-C). A limited phenome-wide association study (PheWAS) was performed for unweighted and weighted PRS and platelet counts, glucose, serum creatinine, serum albumin, serum albumin creatinine ratio, blood urea nitrogen (BUN), uric acid, urine creatinine, urine albumin, and estimated glomerular filtration rate (eGFR). eGFR was calculated using EHR-extracted serum creatinine, age closest to serum creatinine, sex, and race and the CKD-EPI equation (Levey et al., 2009). eGFR was then categorized as stage 1 (≥90 mL/min per 1.73 m^2^), stage 2 (60–89 mL/min per 1.73 m^2^), and stage 3 (<60 mL/min per 1.73 m^2^) for the limited PheWAS. When necessary, laboratory values were transformed as the natural log of the quantitative value plus one per the PAGE I study PheWAS protocol (Pendergrass et al., 2011). All PheWAS tests of association were performed using linear regression except for eGFR where multinomial logistic regression was used to test for association using stage 1 as the referent. All statistical analyses were performed using R version 4.1.0 (R Core Team, 2021) and PLINK 1.90.

## Results

### Study population

A total of 11,521 African American patients were genotyped on the Illumina Metabochip as part of EAGLE BioVU (Crawford et al., 2015). Among these patients, 3,254 were adults with height, weight, and Metabochip genotypes (Table 1). Many (61.16%) were female with an average age of 46.95 years (±15.32 years standard deviation) and an average BMI (31.68 kg/m^2^; ±8.52 kg/m^2^ standard deviation) considered obese. Average pre-medication and post-medication lipid lab values were within the range expected for a general US adult African American population (Cohen et al., 2010; Carroll et al., 2012). Each patient had at least one risk allele for each lipid trait (Fig. S1). On average, African American adult patients had a higher TG PRS (5.95) compared with TC (3.52), HDL-C (3.39) and LDL-C (2.01) (Table 1).

**Table 1:** Study population characteristics for African American adults with at least one lipid lab extracted from the electronic health record. Study population characteristics are based on African American adult (18 years or older) patients who had a mention HDL-C, LDL-C, TG, or TC in the electronic health record (EHR) and who were also genotyped using the Illumina Metabochip. First mention of each lipid lab was extracted and recorded as either a “pre-medication” (free of evidence of concurrent lipid lowering medication) or “post-medication” value as described in Methods and Materials. Body mass index was calculated using the closest height and weight recorded in the EHR compared to the clinic date of the lipid lab. Weighted polygenic risk scores (PRSw) were calculated as described in Methods and Materials.

**Variable**	**Mean (± standard deviation) or %**
Female	61.16%
Age	46.95 (± 15.32) years
Body mass index	31.68 (± 8.52) kg/m^2^
HDL-C	Pre-medication (*n* = 1,464): 53.90 (± 18.16) mg/dL Post-medication (*n* = 428): 50.34 (± 19.39) mg/dL
LDL-C	Pre-medication (*n* = 1,464): 105.23 (± 40.72) mg/dL Post-medication (*n* = 433): 101.82 (± 49.98) mg/dL
Triglycerides	Pre-medication (*n* = 1,535): 115.51 (± 81.13) mg/dL Post-medication (*n* = 441): 134.15 (± 79.56) mg/dL
ln(Triglycerides)	Pre-medication (*n* = 1,535): 4.56 (± 0.60) mg/dL Post-medication (*n* = 441): 4.76 (± 0.53) mg/dL
Total Cholesterol	Pre-medication (*n* = 1,640): 182.20 (± 48.72) mg/dL Post-medication (*n* = 464): 177.88 (± 58.10) mg/dL
HDL-C PRSw	3.39 (± 0.61)
LDL-C PRSw	2.01 (± 0.45)
TG PRSw	5.95 (± 0.51)
TC PRSw	3.52 (± 0.43)

### Lipid trait genetic associations

**Figure 1:**
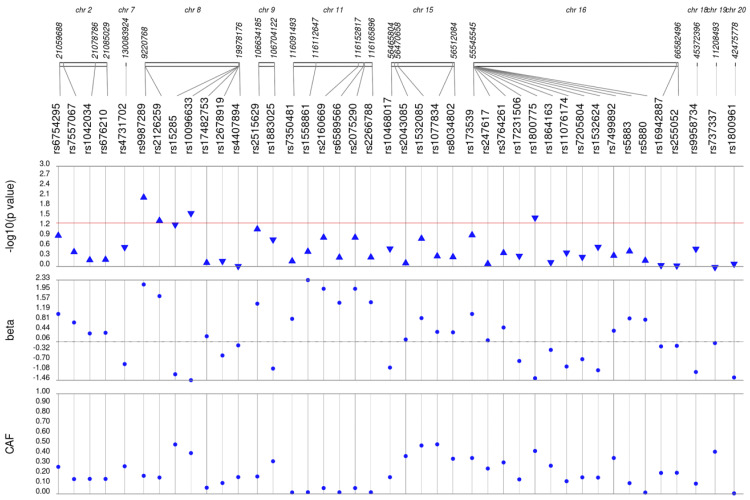
Results of adjusted single SNP tests of associations among African American adults for HDL-C levels, pre-medication. Lipid labs were extracted from EHRs and represent the first mention of the laboratory value free of evidence of concurrent lipid lowering medication usage (“pre-medication”). Each SNP was tested for an association with each pre-medication lipid lab using linear regression assuming an additive genetic model adjusted for age, sex, body mass index, and the first 10 principal components. SNP genomic location is given on the *x*-axis, and *p*-values (−log_10_ transformed) are plotted along the *y*-axis using Synthesis View. The direction of the arrows corresponds to the direction of the beta-coefficient. The significance threshold is indicated by the red line at *p* = 0.05. Also plotted are the betas and the coded allele frequencies (CAF).

**Figure 2:**
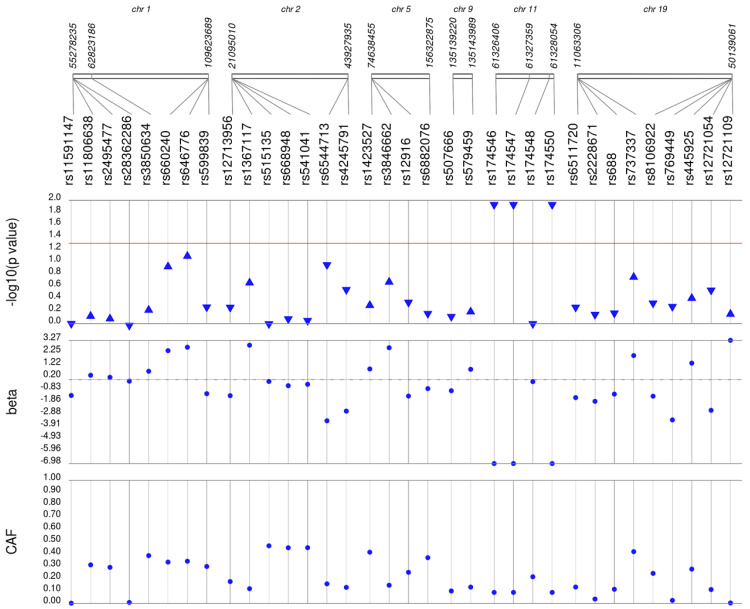
Results of adjusted single SNP tests of associations among African American adults for LDL-C levels, pre-medication. Lipid labs were extracted from EHRs and represent the first mention of the laboratory value free of evidence of concurrent lipid lowering medication usage (“pre-medication”). Each SNP was tested for an association with each pre-medication lipid lab using linear regression assuming an additive genetic model adjusted for age, sex, body mass index, and the first 10 principal components. SNP genomic location is given on the *x*-axis, and *p*-values (−log_10_ transformed) are plotted along the *y*-axis using Synthesis View. The direction of the arrows corresponds to the direction of the beta-coefficient. The significance threshold is indicated by the red line at *p* = 0.05. Also plotted are the betas and the coded allele frequencies (CAF).

**Figure 3:**
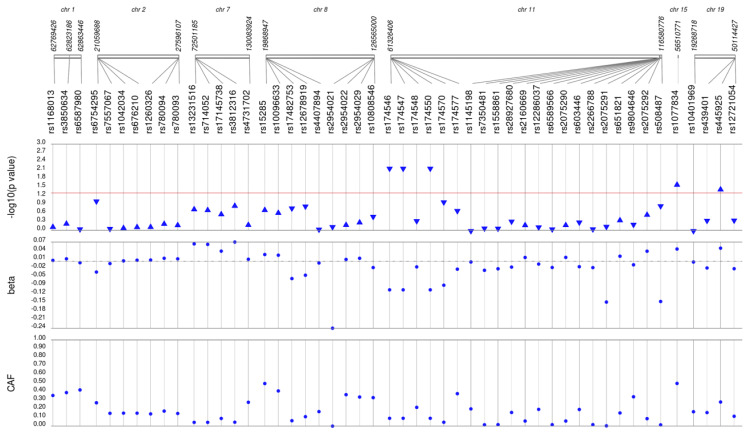
Results of adjusted single SNP tests of associations among African American adults for triglyceride levels, pre-medication. Lipid labs were extracted from EHRs and represent the first mention of the laboratory value free of evidence of concurrent lipid lowering medication usage (“pre-medication”). Each SNP was tested for an association with each pre-medication lipid lab using linear regression assuming an additive genetic model adjusted for age, sex, body mass index, and the first 10 principal components. Triglyceride levels were transformed (natural log) prior to tests of association. SNP genomic location is given on the *x*-axis, and *p*-values (−log_10_ transformed) are plotted along the *y*-axis using Synthesis View. The direction of the arrows corresponds to the direction of the beta-coefficient. The significance threshold is indicated by the red line at *p* = 0.05. Also plotted are the betas and the coded allele frequencies (CAF).

**Figure 4:**
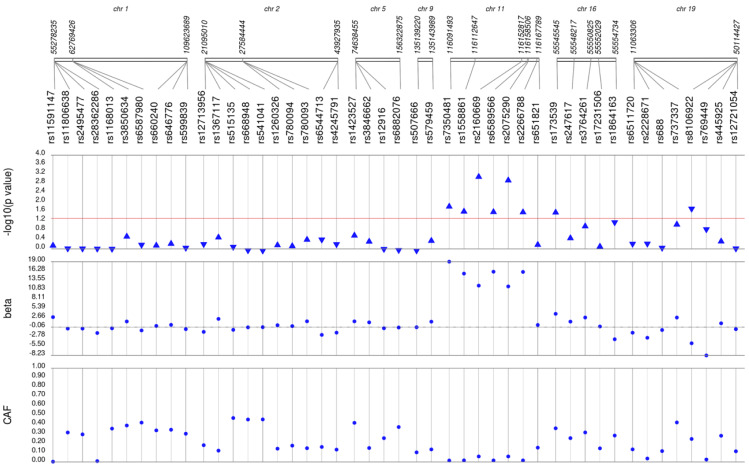
Results of adjusted single SNP tests of associations among African American adults for total cholesterol levels, pre-medication. Lipid labs were extracted from EHRs and represent the first mention of the laboratory value free of evidence of concurrent lipid lowering medication usage (“pre-medication”). Each SNP was tested for an association with each pre-medication lipid lab using linear regression assuming an additive genetic model adjusted for age, sex, body mass index, and the first 10 principal components. SNP genomic location is given on the *x*-axis, and *p*-values (−log_10_ transformed) are plotted along the *y*-axis using Synthesis View. The direction of the arrows corresponds to the direction of the beta-coefficient. The significance threshold is indicated by the red line at *p* = 0.05. Also plotted are the betas and the coded allele frequencies (CAF).

We first performed unadjusted (Fig. S2) and adjusted single SNP tests of association for each lipid trait, stratified by lipid lowering medication exposure as described in **Materials & Methods**. Among African Americans with pre-medication lipid labs, four out of 42 HDL-C, three out of 34 LDL-C, five out of 50 TG, and eight out of 46 TC SNPs were associated with their respective lipid trait at *p* < 0.05 adjusted for sex, age, BMI, and PCs (Figs. 1–4). Compared with the literature and accounting for coded alleles (Hu et al., 2020), all associations identified here at *p* < 0.05 for LDL-C were in the expected directions of effect. Most of the associations identified here for HDL-C were in the opposite direction compared with Hu et al. (2020), with only rs1800775 associated in the expected direction. For transformed triglycerides (rs1077834) and total cholesterol (rs8106922) each, all but one of the associated SNPs were in the expected direction of effect.

Among African Americans with post-medication lipid labs, two out of 42 HDL-C, four out of 34 LDL-C, and one out of 49 TG SNPs were associated with their respective lipid trait at *p* < 0.05 adjusted for sex, age, BMI, and PCs (Fig. S3). None of the SNPs tested were associated with post-medication TC at *p* < 0.05. Overall, fewer associations at *p* < 0.05 were identified among post-medication lipid labs (eight) compared with pre-medication lipid labs (20), most likely due to differences in sample size (Table 1). Despite the smaller sample sizes, three associations were identified among African Americans with post-medication labs but not among African Americans with pre-medication labs: rs1864163 (HDL-C), rs688 (LDL-C), and rs4407894 (transformed TG) (Figs. 1–4 *versus* Fig. S3). Of these three associations, rs1864163 was associated in the opposite direction compared with Hu et al. (2020). Among the five overlapping pre- and post-medication associations, all were consistent in direction of effects with the exception of rs1800775, which was associated with decreased HDL-C levels among African Americans with pre-medication labs but with increased levels among African Americans with post-medication labs.

### Lipid PRS, CVD, and T2D

We calculated unweighted and weighted PRS for each lipid trait using genomic discovery data specific for diverse populations as described in **Materials & Methods**. Among unadjusted and adjusted tests of association, none were associated with their respective lipid traits at *p* < 0.05 (Table 2). All PRS tested here, regardless of significance, were associated with decreased lipid lab values.

To explore the potential clinical utility of lipid PRS, we tested each lipid weighted PRS for association with CVD and T2DM (Table 3). In this African American patient population, 29.5% had one or more CVD event(s) recorded in their EHR (MI (1.7%), heart attack (29.4%). CABG (1.0%), CSA (1.5%)). Similarly, almost one-third of African American adult patients in the present study had T2D (28.4%). We did not detect significant associations between any of the lipid weighted PRS and T2D at *p* < 0.05 (Table 3). For LDL-C PRS, CVD cases had a higher proportion of extreme LDL-C weighted PRS (10.9%) compared with controls (9.6%) (OR = 1.15, 95% Cl [1.01–1.32]; *p* = 0.04). The association was no longer nominally significant when adjusted for LDL-C levels (*p* > 0.05).

**Table 2:** Results of weighted and unweighted polygenic risk score test of associations among African American adults for HDL-C, LDL-C, TG, and TC levels, pre-medication. Both weighted and unweighted polygenic risk scores (PRS) were calculated as described in Materials & Methods for all patients with a lipid lab value. Lipid labs were extracted from EHRs and represent the first mention of the laboratory value free of evidence of concurrent lipid lowering medication usage (“pre-medication”). Tests of association between PRS and their respective lipid labs were performed using linear regression unadjusted (A), adjusted for sex, age, and body mass index (BMI) (B), and adjusted linear regression for sex, age, BMI, and principal components (C). TG levels were transformed (natural log) prior to tests of association.

PRS	HDL-C		LDL-C		TG		TC	
	**Beta** **(SE)**	**p-value**	**Beta** **(SE)**	**p-value**	**Beta** **(SE)**	**p-value**	**Beta** **(SE)**	**p-value**
**A.** **Unadjusted Linear Regression**								
Weighted	−0.0029(0.7626)	0.9970	−3.7776(2.3805)	0.1128	−0.0311 (0.0293)	0.2887	−2.1158 (2.8190)	0.4530
Unweighted	−0.0453 (0.1033)	0.6615	−0.4448(0.2754)	0.1065	−0.0051 (0.0034)	0.1277	−0.1399 ((0.2663))	0.5994
**B.** **Adjusted Linear Regression**								
Weighted	−0.3625(0.7247)	0.6169	−3.7223(2.3595)	0.1149	−0.0306 (0.0280)	0.2747	−1.9797(2.7868)	0.4776
Unweighted	−0.0994(0.0982)	0.3118	−0.4472(0.2731)	0.1017	−0.0050 (0.0032)	0.1199	−0.1284 (0.2632)	0.6256
**C.** **Adjusted Linear Regression + Principal Components**								
Weighted	−0.3102(0.7293)	0.6707	−3.0489 (2.4835)	0.2198	−0.0346 (0.0289)	0.2314	−1.6617 (2.8394)	0.5585
Unweighted	−0.0928(0.0990)	0.3490	−0.3855 (0.2917)	0.1865	−0.0052 (0.0033)	0.1147	−0.1120 (0.2683)	0.6764

**Table 3:** Results from tests of association between weighted polygenic risk scores for lipid traits and cardiovascular disease and type 2 diabetes (T2D). Prevalent CVD in African American adults was determined using International Classification of Diseases (ICD) 9th edition, clinical modification (CM) codes and current procedural terminology (CPT) codes as well as search terms using problems list and clinical notes as described in Materials & Methods. Type 2 diabetes (T2D) case status was determined using an algorithm developed and validated by the eMERGE Network. For both CVD and T2D case and control groups, we determined the proportion of African American patients in the upper 90th percentile of each lipid trait weighted polygenic risk score (PRSw) as well as the proportion of patients in the lower 90th percentiles. Tests of association were performed as a 2 × 2 table, and shown are the resulting odds ratios, 95% confidence intervals (CI), and pvalues.

wPRS	Outcome and sample sizes	% Cases High PRSw/ % Cases Lower PRSw	% Controls High PRSw/ % Controls Lower PRSw	Odds Ratio (95% CI)	*P*-value
HDL-C	CVD n_cases_= 3,248 n_controls_= 7,759	10.7/89.3	9.7/90.3	1.12 (0.98, 1.29)	0.094
	T2D n_cases_= 1,330 n_controls_= 4,053	10.5/89.5	9.8/90.2	1.07 (0.87, 1.31)	0.53
LDL-C	CVD n_cases_= 3,244 n_controls_= 7,743	10.9/89.1	9.6/90.4	1.15 (1.01, 1.32)	0.04
	T2D n_cases_= 1,330 n_controls_= 4,031	9.4/90.6	10.2/89.8	0.91 (0.73, 1.13)	0.43
TG	CVD n_cases_= 3,154 n_controls_= 7,522	9.8/90.2	10.1/89.9	0.97 (0.84, 1.12)	0.72
	T2D n_cases_= 1,297 n_controls_= 3,952	8.6/91.4	10.4/89.6	0.81 (0.65, 1.01)	0.06
TC	CVD n_cases_= 3,231 n_controls_= 7,712	10.2/89.8	9.9/90.1	1.03 (0.90, 1.19)	0.62
	T2D n_cases_= 1,323 n_controls_= 4,016	9.1/90.9	10.3/89.7	0.88 (0.71, 1.09)	0.27

### Limited PheWAS

To identify possible pleiotropic relationships between genetic determinants of lipid traits and other commonly ordered clinical labs, we performed a limited PheWAS for each lipid PRS and EHR-extracted labs related to biochemistry, liver, and kidney functions. Nominal associations (*p* < 0.05) were identified for HDL-C PRS and albumin creatinine ratio; serum creatinine and LDL-C and TC PRS; and stage 2 eGFR and TC PRS (Table 4). However, after adjusting significance thresholds for multiple testing (four lipid trait PRS ×10 labs), none of the limited PheWAS associations remained significant.

**Table 4:** Limited phenome-wide association study for lipid trait weighted PRS and common clinical labs in African American adult patients. Lipid trait weighted polygenic risk scores (wPRS) were calculated as described in Materials & Methods. Lab values were transformed (*) when necessary, using log10(x+1). All tests of association were performed using linear regression for each lipid trait PRSw and each quantitative trait lab with the exception of estimated glomerular filtration rate (eGFR). We categorized eGFR where patients with eGFR ≥ 90 mL/min/1.73m2 are considered within normal limits and the reference group here (REF). Patients with eGFR 60-90 and <60 were considered stage 2 and stage 3, respectively. Stage 2 and stage 3 were compared with stage 1, respectively, using logistic regression. Shown are test of association betas, standard errors (SE), and p-values by lipid trait PRSw and organized by clinical lab category.

Weighted PRS								
	**HDL-C**		**LDL-C**		**TG**		**TC**	
	**Beta** **(SE)**	**p-value**	**Beta** **(SE)**	**p-value**	**Beta** **(SE)**	**p-value**	**Beta** **(SE)**	**p-value**
**Biochemistry**								
**Platelet Count**	0.1469 (1.4862)	0.9213	−2.2072 (2.1179)	0.2974	0.4775 (1.8077)	0.7917	−2.1575 (2.1881)	0.3242
**Glucose**	1.5967 (0.8484)	0.0599	−0.3872 (1.2069)	0.7484	0.2874 (1.0440)	0.7831	−0.0652 (1.2427)	0.9582
**Serum Creatinine **	0.0067 (0.0053)	0.2057	0.0161 (0.0076)	0.0340	0.0053 (0.0065)	0.4122	0.0203(0.0078)	0.0092
**Liver**								
Serum Albumin *	−0.0004 (0.0006)	0.5824	0.0001 (0.0009)	0.8930	−0.0014 (0.0008)	0.0865	0.0001 (0.0009)	0.8876
**Kidney**								
Albumin Creatinine Ratio *	0.0952 (0.0344)	0.0057	−0.0190 (0.0494)	0.7008	0.0229 (0.0430)	0.5945	0.0156 (0.0508)	0.7596
Blood Urea Nitrogen *	0.0070 (0.0041)	0.0852	0.0039 (0.0058)	0.4969	0.0036 (0.0050)	0.4778	0.0088 (0.0059)	0.1390
**Uric acid**	−0.0780 (0.0664)	0.2403	0.1171 (0.0956)	0.2207	−0.0313 (0.0839)	0.7094	0.1376 (0.0988)	0.1640
Urine Creatinine *	0.0401 (0.0382)	0.2962	−0.0018 (0.0508)	0.9714	0.0600 (0.0498)	0.2307	0.0273 (0.0575)	0.6360
Urine Albumin *	0.0002 (0.0205)	0.9934	−0.0332 (0.0282)	0.2397	0.0348 (0.0250)	0.1653	−0.0152 (0.0293)	0.6038
**eGFR**								
**Stage 2**	−0.0136 (0.0437)	0.7552	0.1095 (0.0619)	0.0770	0.0557 (0.0537)	0.2995	0.1365 (0.0638)	0.0324
**Stage 3**	0.0745 (0.0468)	0.1117	0.04967 (0.0670)	0.4577	0.0568 (0.0580)	0.3275	0.0744 (0.0689)	0.2808

## Discussion

PRS have the potential to identify patients at-risk for developing health outcomes early in the disease process (Torkamani, Wineiger & Topol, 2018; Kullo et al., 2022). Given genomic discovery for common human diseases remains biased by genetic ancestry (Sirugo, Williams & Tishkoff, 2019; Peterson et al., 2019), there is much interest in assessing the generalizability of current PRS in diverse populations (Wang et al., 2022). In this study we calculated multi-population PRS for HDL-C, LDL-C, TG, and TC using (1) specific genetic variants and (2) their respective weights associated with these traits identified in a diverse population that includes a substantial African American sample (Hu et al., 2020) and applied them to an independent African American patient population from a biobank linked to real-world clinical data. Overall, we did not observe strong associations between unweighted or weighted lipid PRS for the lipid traits tested here or for T2D and other common clinical labs. We did, however, observe a nominal association between PRS_LDL−C_ and CVD.

The lack of associations between lipid PRS and their respective lipid traits was somewhat unexpected as previous reports have demonstrated strong associations between lipid traits and genetic or polygenic risk scores. In contrast to the present study, lipid trait PRS in Biobank Japan were strongly associated to their respective lipid labs and in the expected directions (Tam et al., 2021). Similarly, lipid PRS developed in the diverse Multi-Ethnic Study of Atherosclerosis (MESA) using variants discovered in European-descent GWAS were strongly associated with their respective lipid traits in the expected direction for all populations except for TG in African Americans (Johnson et al., 2015). In a recent generalization study, European-derived LDL-C, HDL-C, and TG PRS were also strongly associated with their respective lipid traits in populations from Europe, Asia, and Africa (Kuchenbaecker et al., 2019). Strong associations between European-derived LDL-C, HDL-C, TG, and TC PRS have also been reported for a study of adolescents from the Netherlands (Xie et al., 2020).

Literature specific to lipid trait PRS derived from (all or in part) and applied to African Americans is limited. A recent assessment LDL-C, HDL-C, TG, and TC PRS derived from European, multi-ancestral, and sub-Saharan African discovery meta-analyses all demonstrated strong associations in the expected directions for their respective lipid traits in an independent sample of 7,103 participants from the Africa Wits-INDEPTH Partnership for Genomic Studies (AWI-Gen) cohort (Choudhury et al., 2022). Of note is a recent study from BioVU where chip-wide LDL-C, HDL-C, and TG PRS were developed and applied to African American patients who largely overlap with the present study (Dennis et al., 2021). Strong associations were observed between each PRS and its respected lipid trait (Dennis et al., 2021). Consistent with the larger BioVU study, we did not identify strong associations between lipid trait PRS and common clinical labs in the limited PheWAS (Dennis et al., 2021). Differences between the larger BioVU study and the present study are numerous, including differences in sample size, genotyping array, computable phenotyping approach, year of clinical data extraction, and methods used to calculate PRS.

As the two studies accessing BioVU demonstrate, direct comparison of PRS associations in the literature is challenging for multiple reasons. First and foremost, there is no consensus on how to calculate or define PRS. As of this writing, the Polygenic Risk Score Catalog (Lambert et al., 2021) reports 66 PRS available in the literature for lipid traits, including 27,10, 9, and 7 for LDL-C, HDL-C, TG, and TC, respectively. PRS, even for the same trait (*e.g.*, LDL-C), differ by the number of variants included and how variants are chosen. PRS also differ by the discovery data from which they draw both variants and weights, much of which is still focused on European-descent populations. Finally, there are no standards on the evaluation and reporting of PRS performance. In the present study, we selected index variants associated with lipid traits in a diverse population that includes African Americans and calculated unweighted and weighted PRS using the absolute values of the betas reported in the discovery study. We applied the PRS to an independent sample of African American patients and reported the betas and *p*-values from unadjusted and adjusted tests of association. Collectively, the comparison challenges we experienced here highlight the need to develop approaches to standardize PRS being developed for implementation in a clinical setting to benefit patients (Wand, Knowles & Clarke, 2021).

While we did not observe strong associations between the PRS and their lipid traits, we did observe a nominal association between PRS_LDL−C_ and CVD. Our results are consistent with an early PRS_LDL−C_ applied to the Malmö Diet and Cancer Study, a prospective study of European-descent participants where PRS_LDL−C_ constructed from nine SNPs predicted risk of first CVD event independent of known risk factors (Kathiresan et al., 2008). Here, we observed a greater proportion of CVD cases among the African American patients in the 90th PRS_LDL−C_ percentile compared with African American patients in the same 90th percentile without CVD. Despite differences in genetic ancestry, these data are consistent with PRS_LDL_ associations and incident CVD recently observed in the UK Biobank (Tromp et al., 2022). The trend observed here is also consistent with data in Biobank Japan, which suggested associations between coronary heart disease among T2D patients and PRS_LDL_, PRS_TG_, and PRS_TC_ (Tam et al., 2021). To the best of our knowledge, no similar studies yet exist that are specific to African Americans with CVD or lipid trait PRS examined for relationships to T2D risk in any population.

The present study has several limitations. The sample size is small, resulting in low statistical power. With an initial dataset of almost 12,000 African Americans, the dataset was reduced to ∼2,000 per lipid trait due to missing data and data cleaning as is characteristic of clinical data. While previous published assessments suggest the computable phenotyping approaches employed here result in high quality, research grade variables (Crawford et al., 2015; Crawford et al., 2018; Dumitrescu et al., 2015b; Dumitrescu et al., 2015c; Dumitrescu et al., 2015a; Goodloe et al., 2017), the CVD variable is a mix of prevalent and incident cases, limiting the assessment of PRS risk. Other limitations are related to PRS calculation. We drew from the genotyped Metabochip and selected index variants associated with lipid traits regardless of linkage disequilibrium. Prevailing PRS methods discourage the inclusion of genetic variants in strong linkage disequilibrium, which can lead to artificial score inflation. The mostly null associations reported here suggest score inflation was not substantial. In addition to the already-noted small sample sizes, the mostly null associations observed here may be a result of an imperfect ancestral match (Privé et al., 2022) between the diverse and heterogeneous (Choudhury et al., 2020) populations of Hu et al. (2020) and the African American patient population studied here. While we based PRS calculations on data that include African Americans, we acknowledge that PRS best practices for diverse populations are actively being developed and evaluated (Graham et al., 2021).

## Conclusions

Despite the challenges of sample size, this is one of the few studies of multi-population lipid PRS developed from a genomic discovery effort that includes African Americans and applied to an independent African American patient population. In general, larger, prospective diverse cohorts are needed to assess clinical utility of PRS for a variety of common clinic outcomes.

##  Supplemental Information

10.7717/peerj.14910/supp-1 Supplemental Information 1 Results of unadjusted single SNP tests of associations among African American adults for (A) HDL-C, (B) LDL-C, (C) TG, and (D) TC levels, pre-medication Lipid labs were extracted from EHRs and represent the first mention of the laboratory value free of evidence of concurrent lipid lowering medication usage (“pre-medication”). Each SNP was tested for an association with each pre-medication lipid lab using linear regression assuming an additive genetic model. SNP genomic location is given on the *x*-axis, and *p*-values (−log_10_ transformed) are plotted along the *y*-axis using Synthesis View. The direction of the arrows corresponds to the direction of the beta-coefficient. The significance threshold is indicated by the red line at *p* = 0.05. Also plotted are the betas and the coded allele frequencies (CAF).

10.7717/peerj.14910/supp-2 Supplemental Information 2 Results of adjusted single SNP tests of associations among African American adults for (A) HDL-C, (B) LDL-C, (C) TG, and (D) TC levels, post-medication Lipid labs were extracted from EHRs and represent the first mention of the laboratory value with evidence of concurrent lipid lowering medication usage (“post-medication”). Each SNP was tested for an association with each post-medication lipid lab using linear regression assuming an additive genetic model adjusted for age, sex, body mass index, and the first 10 principal components. Triglyceride levels were transformed (natural log) prior to tests of association. SNP genomic location is given on the *x*-axis, and *p*-values (−log_10_ transformed) are plotted along the *y*-axis using Synthesis View. The direction of the arrows corresponds to the direction of the beta-coefficient. The significance threshold is indicated by the red line at *p* = 0.05. Also plotted are the betas and the coded allele frequencies (CAF).

10.7717/peerj.14910/supp-3 Supplemental Information 3 Results of adjusted single SNP tests of associations among African American adults for (A) HDL-C, (B) LDL-C, (C) TG, and (D) TC levels, post-medication Lipid labs were extracted from EHRs and represent the first mention of the laboratory value with evidence of concurrent lipid lowering medication usage (“post-medication”). Each SNP was tested for an association with each post-medication lipid lab using linear regression assuming an additive genetic model adjusted for age, sex, body mass index, and the first 10 principal components. Triglyceride levels were transformed (natural log) prior to tests of association. SNP genomic location is given on the *x*-axis, and *p*-values (−log_10_ transformed) are plotted along the *y*-axis using genome build NCBI36/hg18 in Synthesis View. The direction of the arrows corresponds to the direction of the beta-coefficient. The significance threshold is indicated by the red line at *p* = 0.05. Also plotted are the betas and the coded allele frequencies (CAF).

10.7717/peerj.14910/supp-4 Supplemental Information 4 SNPs associated with lipid traits used to calculate population appropriate polygenic risk scores We identified SNPs genotyped on the Metabochip that were reported as associated at *p* ≤ 5.0 × 10^−8^ with high density lipoprotein cholesterol (HDL-C), low density lipoprotein cholesterol (LDL-C), total cholesterol (TC), and triglycerides (TG) by (Hu et al., 2020). Shown per variant are chromosomal location, rs number, associated lipid trait, and genetic effect size.

10.7717/peerj.14910/supp-5 Supplemental Information 5 Synthesis View input files for HDL-C, LDL-C, ln(TG), and TC tests of association, pre-medication Results of tests of association are given here formatted for Synthesis View. These summary statistics include SNP ID (rs number), chromosome (chr) and position (NCBI36/hg18), *p*-value (pval), beta as the effect size (es), and coded allele frequency (caf). Summary statistics are given for unadjusted and adjusted tests of association between each SNP and lipid trait. Adjusted models are models adjusted for age, sex, body mass index, and principal components.

## Additional Information and Declarations

### Data Availability

The following information was supplied regarding data availability: The NHGRI PAGE EAGLE MetaboChip study is available at dbGaP. - The individual-level data are available for download by authorized investigators: https://view.ncbi.nlm.nih.gov/dbgap-controlled
- Data dictionaries, variable summaries, and documents are available on the dbGaP FTP site: https://ftp.ncbi.nlm.nih.gov/dbgap/studies/phs002767/phs002767.v1.p1
- The public summary-level phenotype data may be browsed at the dbGaP study report page: https://www.ncbi.nlm.nih.gov/projects/gap/cgi-bin/study.cgi?study_id=phs002767.v1.p1
The NIH Genomic Data Sharing (GDS) Policy sets the expectation that the informed consent under which the data or samples were collected is the basis for determining the appropriateness of sharing data through unrestricted-access databases or NIH designated controlled-access data repositories (e.g.,dbGaP).
