# Supplementary material for: Assessment of multi-population polygenic risk scores for lipid traits in African Americans
Source: PeerJ. 2023 May 16;11:e14910. doi: 10.7717/peerj.14910 (PMC10198155; doi:10.7717/peerj.14910)

Supplementary Figure 1. Distributions of HDL-C, LDL-C, TG, and TC polygenic risk scores among African American adults, unweighted and weighted. Unweighted and weighted population-appropriate PRSs for HDL-C, LDL-C, TG, and TC were calculated for each patient based on published summary statistics for diverse populations from the PAGE Study (Hu et al., 2020). As described in Methods & Materials, we calculated unweighted PRSs for each patient and each lipid trait by counting the number of risk alleles for 42, 34, 50, and 46 HDL-C, LDL-C, TG, and TC-associated SNPs, respectively. PRSs were weighted using the absolute value of the betas for the corresponding associations published by the PAGE Study (Hu et al., 2020).

A) HDL-C, unweighted

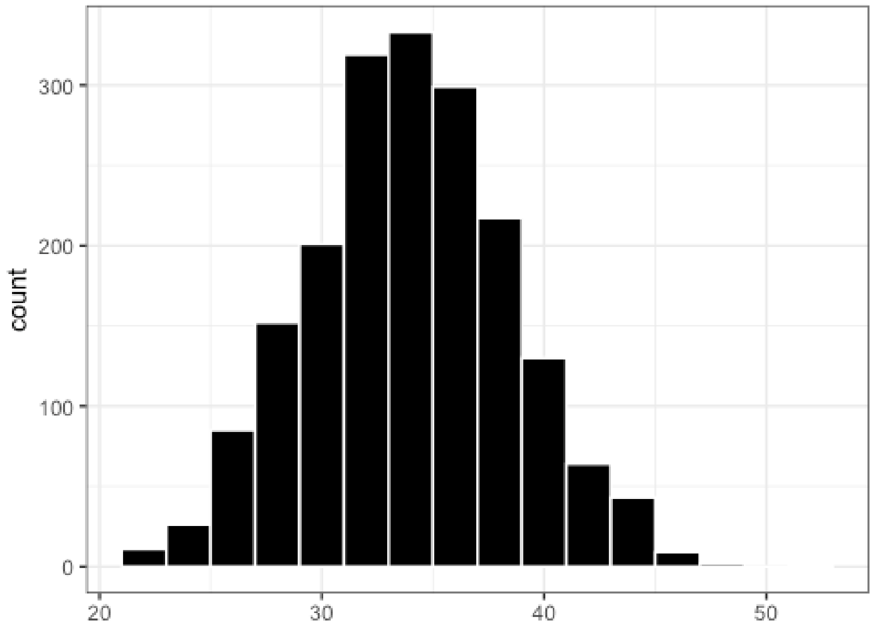

B) LDL-C, unweighted

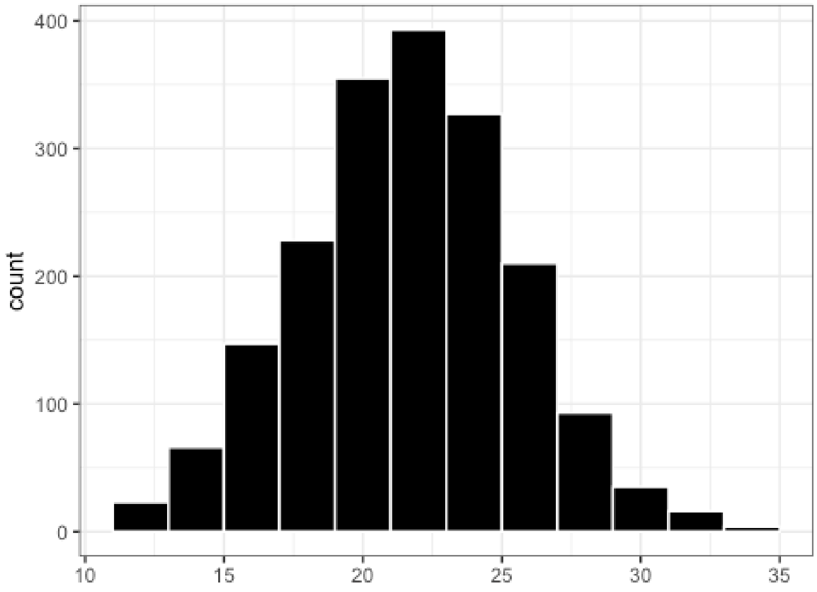

C) TG, unweighted

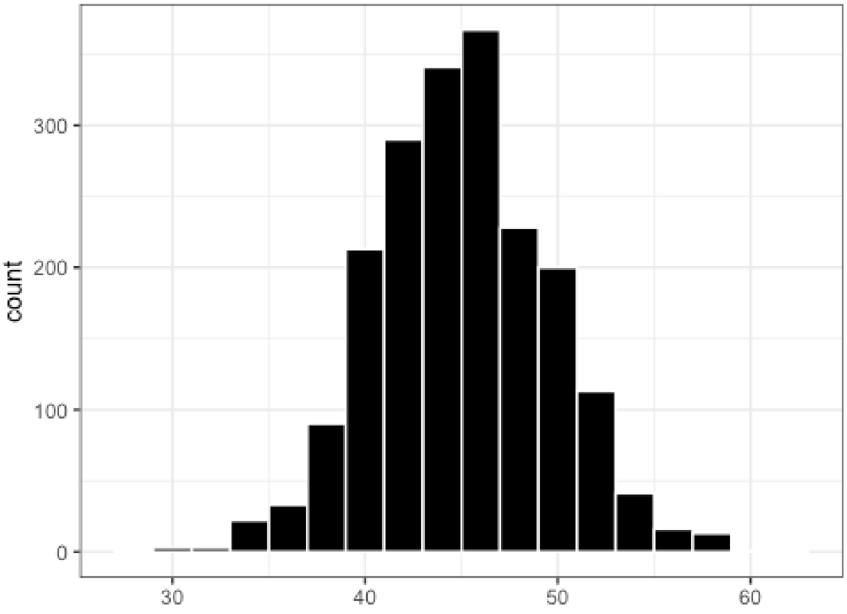

D) TC, unweighted

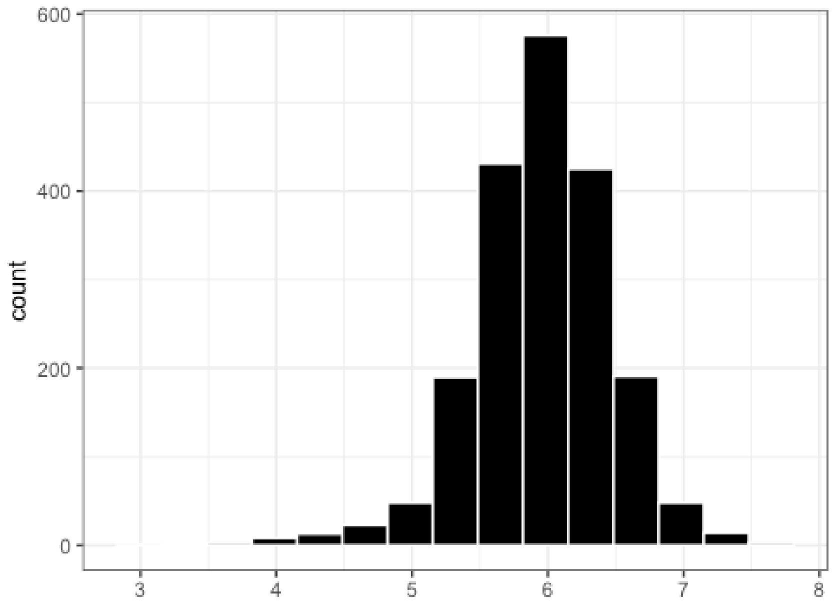

E) HDL-C, weighted

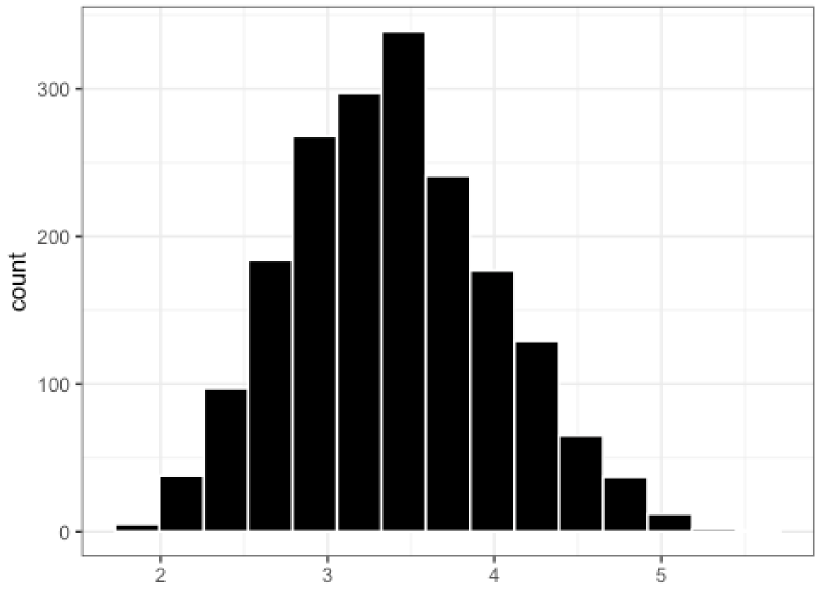

F) LDL-C, weighted

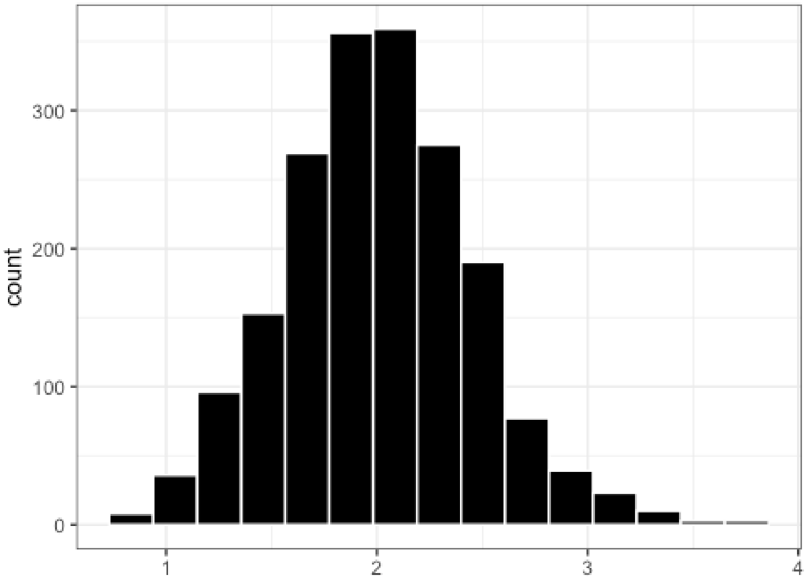

G) TG, weighted

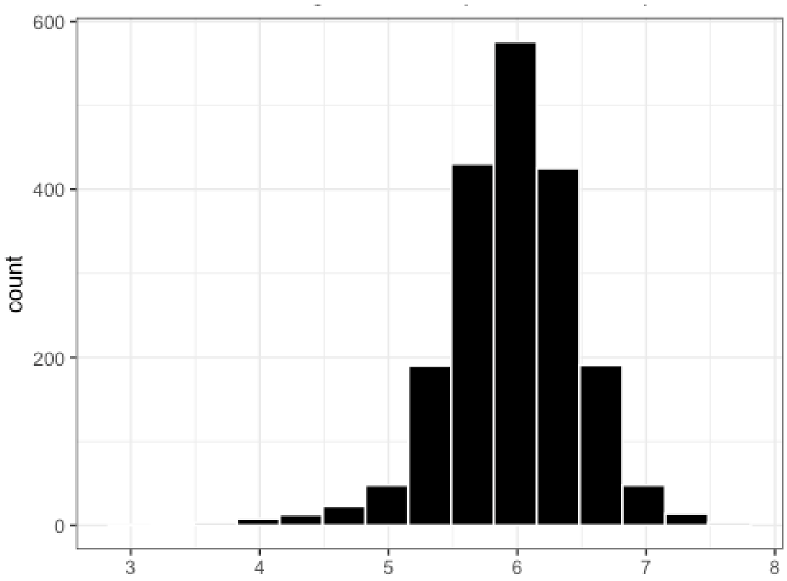

H) TC, weighted

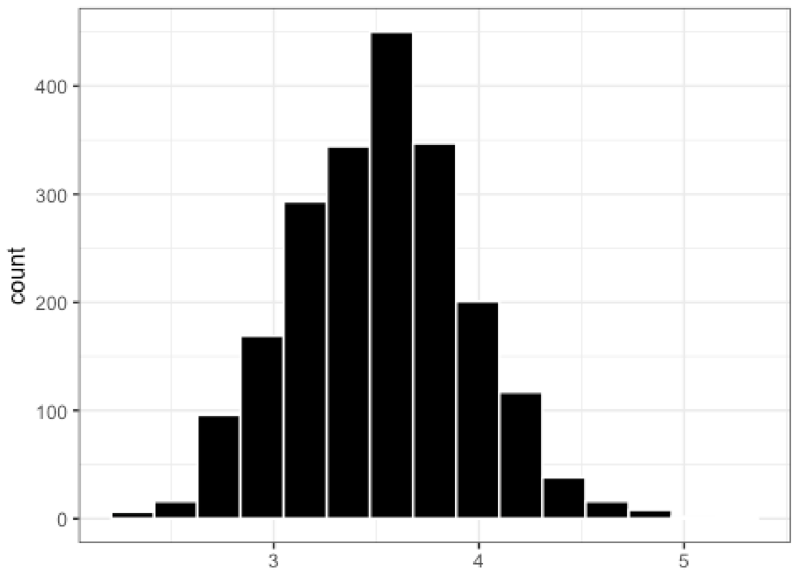

Supplement: Supplemental Information 1 — Lipid labs were extracted from EHRs and represent the first mention of the laboratory value free of evidence of concurrent lipid lowering medication usage (“pre-medication”). Each SNP was tested for an association with each pre-medication lipid lab using linear regression assuming an additive genetic model. SNP genomic location is given on the x-axis, and p-values (−log10 transformed) are plotted along the y-axis using Synthesis View. The direction of the arrows corresponds to the direction of the beta-coefficient. The significance threshold is indicated by the red line at p = 0.05. Also plotted are the betas and the coded allele frequencies (CAF). [file peerj-11-14910-s001.pdf]
