# Supplementary material for: Assessment of multi-population polygenic risk scores for lipid traits in African Americans
Source: PeerJ. 2023 May 16;11:e14910. doi: 10.7717/peerj.14910 (PMC10198155; doi:10.7717/peerj.14910)
Supplement: Supplemental Information 2 — Lipid labs were extracted from EHRs and represent the first mention of the laboratory value with evidence of concurrent lipid lowering medication usage (“post-medication”). Each SNP was tested for an association with each post-medication lipid lab using linear regression assuming an additive genetic model adjusted for age, sex, body mass index, and the first 10 principal components. Triglyceride levels were transformed (natural log) prior to tests of association. SNP genomic location is given on the x-axis, and p-values (−log10 transformed) are plotted along the y-axis using Synthesis View. The direction of the arrows corresponds to the direction of the beta-coefficient. The significance threshold is indicated by the red line at p = 0.05. Also plotted are the betas and the coded allele frequencies (CAF). [file peerj-11-14910-s002.pdf]

Supplementary Figure 2. Results of unadjusted single SNP tests of associations among African American adults for a) HDL-C, b) LDL-C, c) TG, and d) TC levels, pre-medication. Lipid labs were extracted from EHRs and represent the first mention of the laboratory value free of evidence of concurrent lipid lowering medication usage ("pre-medication"). Each SNP was tested for an association with each pre-medication lipid lab using linear regression assuming an additive genetic model. SNP genomic location is given on the x-axis, and p-values (-$\log_{10}$ transformed) are plotted along the y-axis using genome build NCBI36/hg18 in Synthesis View. The direction of the arrows corresponds to the direction of the beta-coefficient. The significance threshold is indicated by the red line at p=0.05. Also plotted are the betas and the coded allele frequencies (CAF).

A.

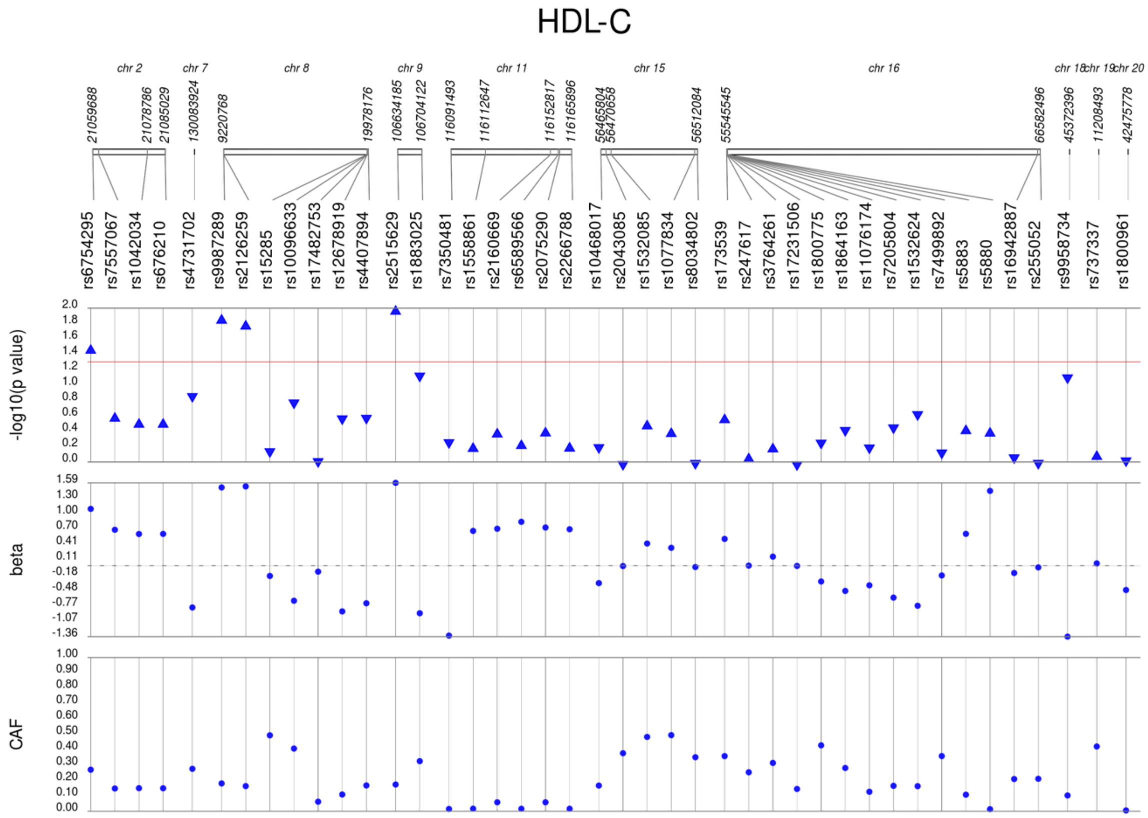
HDL-C
chr 2
chr 7
chr 8
chr 9
chr 11
chr 15
chr 16
chr 18
chr 19
chr 20
-log10(p value)
beta
CAF
rs6754295
rs7557067
rs1042034
rs676210
rs4731702
rs9987289
rs2126259
rs15285
rs10096633
rs17482753
rs12678919
rs4407894
rs2515629
rs1883025
rs7350481
rs1558861
rs2160669
rs6589566
rs2075290
rs2266788
rs10468017
rs2043085
rs1532085
rs1077834
rs8034802
rs173539
rs247617
rs3764261
rs17231506
rs1800775
rs1864163
rs11076174
rs7205804
rs1532624
rs7499892
rs5883
rs5880
rs16942887
rs255052
rs9958734
rs737337
rs1800961

B.

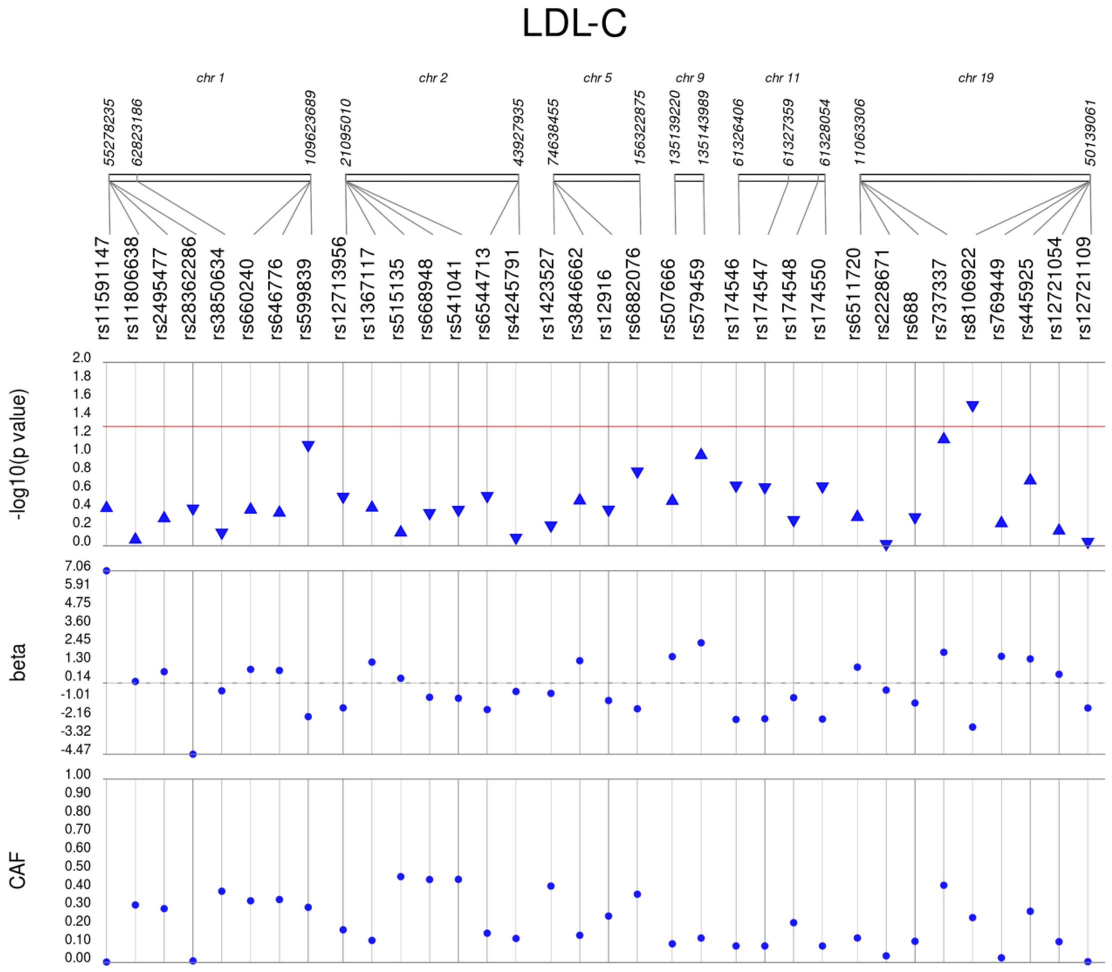
LDL-C
chr 1
chr 2
chr 5
chr 9
chr 11
chr 19
55278235
62823186
109623689
21095010
43927935
74638455
156322875
135139220
135143989
61326406
61327359
61328054
11063306
50139061
rs11591147
rs11806638
rs2495477
rs28362286
rs3850634
rs660240
rs646776
rs599839
rs12713956
rs1367117
rs515135
rs668948
rs541041
rs6544713
rs4245791
rs1423527
rs3846662
rs12916
rs6882076
rs507666
rs579459
rs174546
rs174547
rs174548
rs174550
rs6511720
rs2228671
rs688
rs737337
rs8106922
rs769449
rs445925
rs12721054
rs12721109
-log10(p value)
2.0
1.8
1.6
1.4
1.2
1.0
0.8
0.6
0.4
0.2
0.0
beta
7.06
5.91
4.75
3.60
2.45
1.30
0.14
-1.01
-2.16
-3.32
-4.47
CAF
1.00
0.90
0.80
0.70
0.60
0.50
0.40
0.30
0.20
0.10
0.00

C.

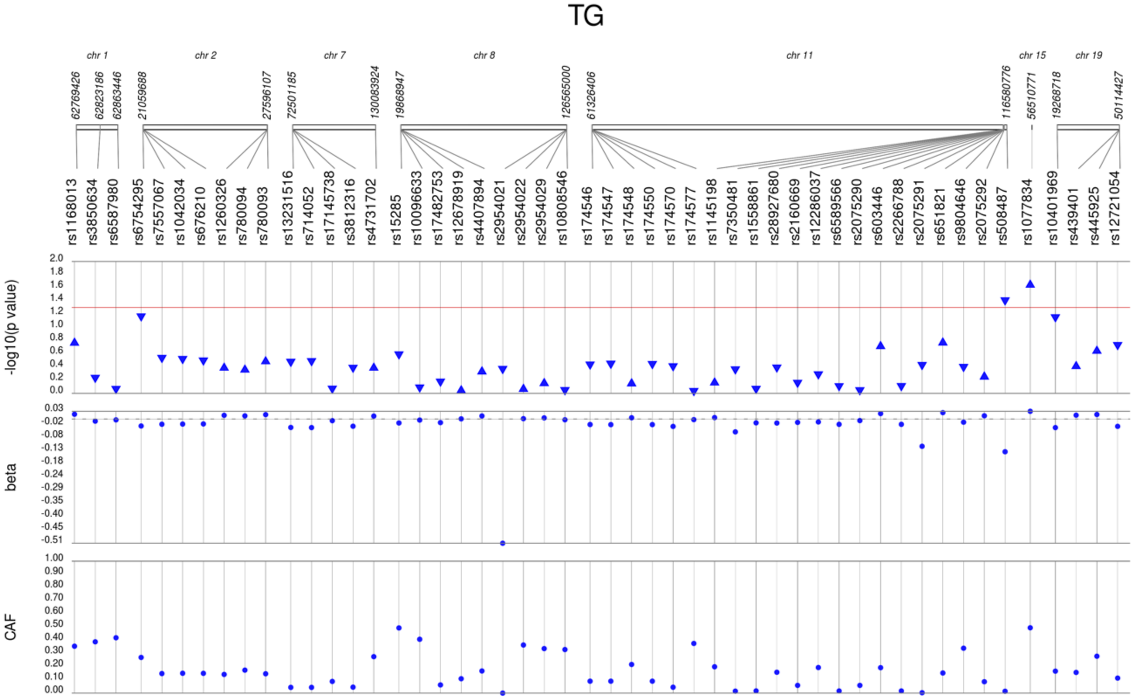
TG
chr 1
chr 2
chr 7
chr 8
chr 11
chr 15
chr 19
62769426
62823186
62863446
21059688
27596107
72501185
130083924
19868947
126565000
61326406
116580776
56510771
19268718
50114427
rs1168013
rs3850634
rs6587980
rs6754295
rs7557067
rs1042034
rs676210
rs1260326
rs780094
rs780093
rs13231516
rs714052
rs17145738
rs3812316
rs4731702
rs15285
rs10096633
rs17482753
rs12678919
rs4407894
rs2954021
rs2954022
rs2954029
rs10808546
rs174546
rs174547
rs174548
rs174550
rs174570
rs174577
rs1145198
rs7350481
rs1558861
rs28927680
rs2160669
rs12286037
rs6589566
rs2075290
rs603446
rs2266788
rs2075291
rs651821
rs9804646
rs2075292
rs508487
rs1077834
rs10401969
rs439401
rs445925
rs12721054
-log10(p value)
beta
CAF

D.

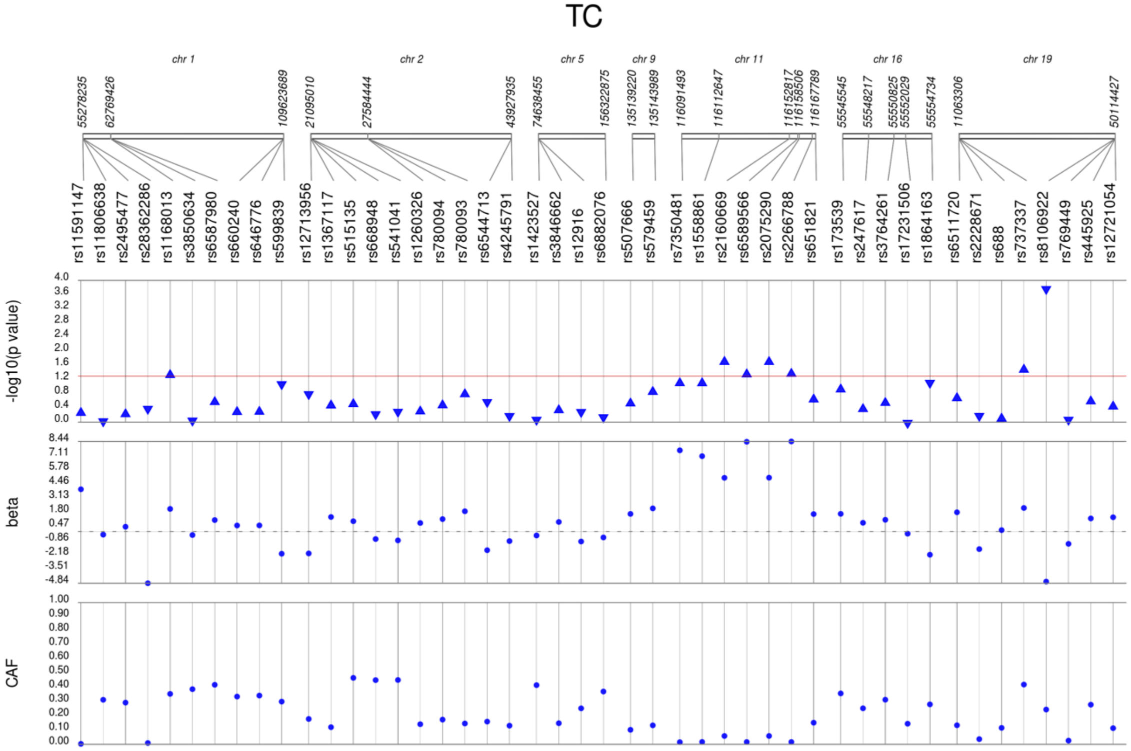
TC
chr 1
chr 2
chr 5
chr 9
chr 11
chr 16
chr 19
55278235
62769426
109623689
21095010
27584444
43927935
74638455
156322875
135139220
135143989
116091493
116112647
116152817
116158506
116167789
55545545
55548217
55550825
55552029
55554734
11063306
50114427
rs11591147
rs11806638
rs2495477
rs28362286
rs1168013
rs3850634
rs6587980
rs660240
rs646776
rs599839
rs12713956
rs1367117
rs515135
rs668948
rs541041
rs1260326
rs780094
rs780093
rs6544713
rs4245791
rs1423527
rs3846662
rs12916
rs6882076
rs507666
rs579459
rs7350481
rs1558861
rs2160669
rs6589566
rs2075290
rs2266788
rs651821
rs173539
rs247617
rs3764261
rs17231506
rs1864163
rs6511720
rs2228671
rs688
rs737337
rs8106922
rs769449
rs445925
rs12721054
-log10(p value)
4.0
3.6
3.2
2.8
2.4
2.0
1.6
1.2
0.8
0.4
0.0
beta
8.44
7.11
5.78
4.46
3.13
1.80
0.47
-0.86
-2.18
-3.51
-4.84
CAF
1.00
0.90
0.80
0.70
0.60
0.50
0.40
0.30
0.20
0.10
0.00
