# Supplementary material for: Assessment of multi-population polygenic risk scores for lipid traits in African Americans
Source: PeerJ. 2023 May 16;11:e14910. doi: 10.7717/peerj.14910 (PMC10198155; doi:10.7717/peerj.14910)
Supplement: Supplemental Information 3 — Lipid labs were extracted from EHRs and represent the first mention of the laboratory value with evidence of concurrent lipid lowering medication usage (“post-medication”). Each SNP was tested for an association with each post-medication lipid lab using linear regression assuming an additive genetic model adjusted for age, sex, body mass index, and the first 10 principal components. Triglyceride levels were transformed (natural log) prior to tests of association. SNP genomic location is given on the x-axis, and p-values (−log10 transformed) are plotted along the y-axis using genome build NCBI36/hg18 in Synthesis View. The direction of the arrows corresponds to the direction of the beta-coefficient. The significance threshold is indicated by the red line at p = 0.05. Also plotted are the betas and the coded allele frequencies (CAF). [file peerj-11-14910-s003.pdf]

Supplementary Figure 3. Results of adjusted single SNP tests of associations among African American adults for a) HDL-C, b) LDL-C, c) TG, and d) TC levels, post-medication. Lipid labs were extracted from EHRs and represent the first mention of the laboratory value with evidence of concurrent lipid lowering medication usage ("post-medication"). Each SNP was tested for an association with each post-medication lipid lab using linear regression assuming an additive genetic model adjusted for age, sex, body mass index, and the first 10 principal components. Triglyceride levels were transformed (natural log) prior to tests of association. SNP genomic location is given on the x-axis, and p-values ($-\log_{10}$ transformed) are plotted along the y-axis using genome build NCBI36/hg18 in Synthesis View. The direction of the arrows corresponds to the direction of the beta-coefficient. The significance threshold is indicated by the red line at p=0.05. Also plotted are the betas and the coded allele frequencies (CAF).

A.

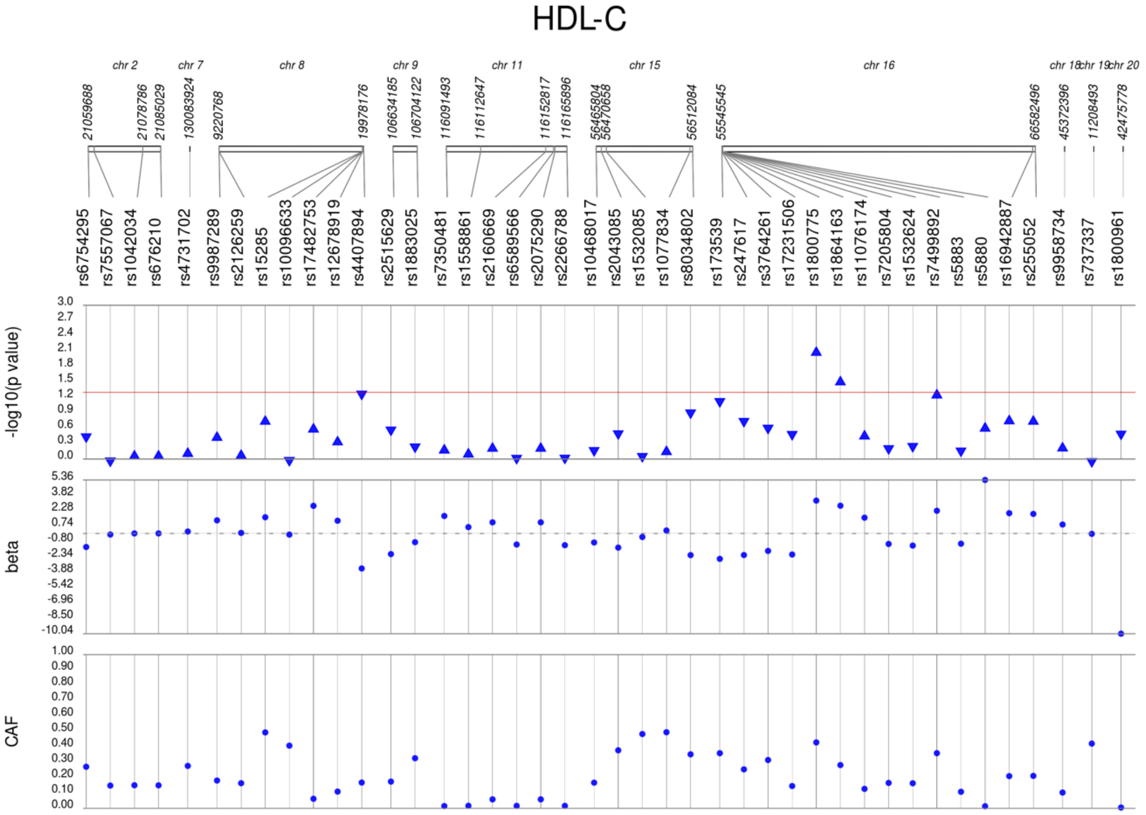

HDL-C
chr 2
chr 7
chr 8
chr 9
chr 11
chr 15
chr 16
chr 18
chr 19
chr 20
-log10(p value)
beta
CAF

B.

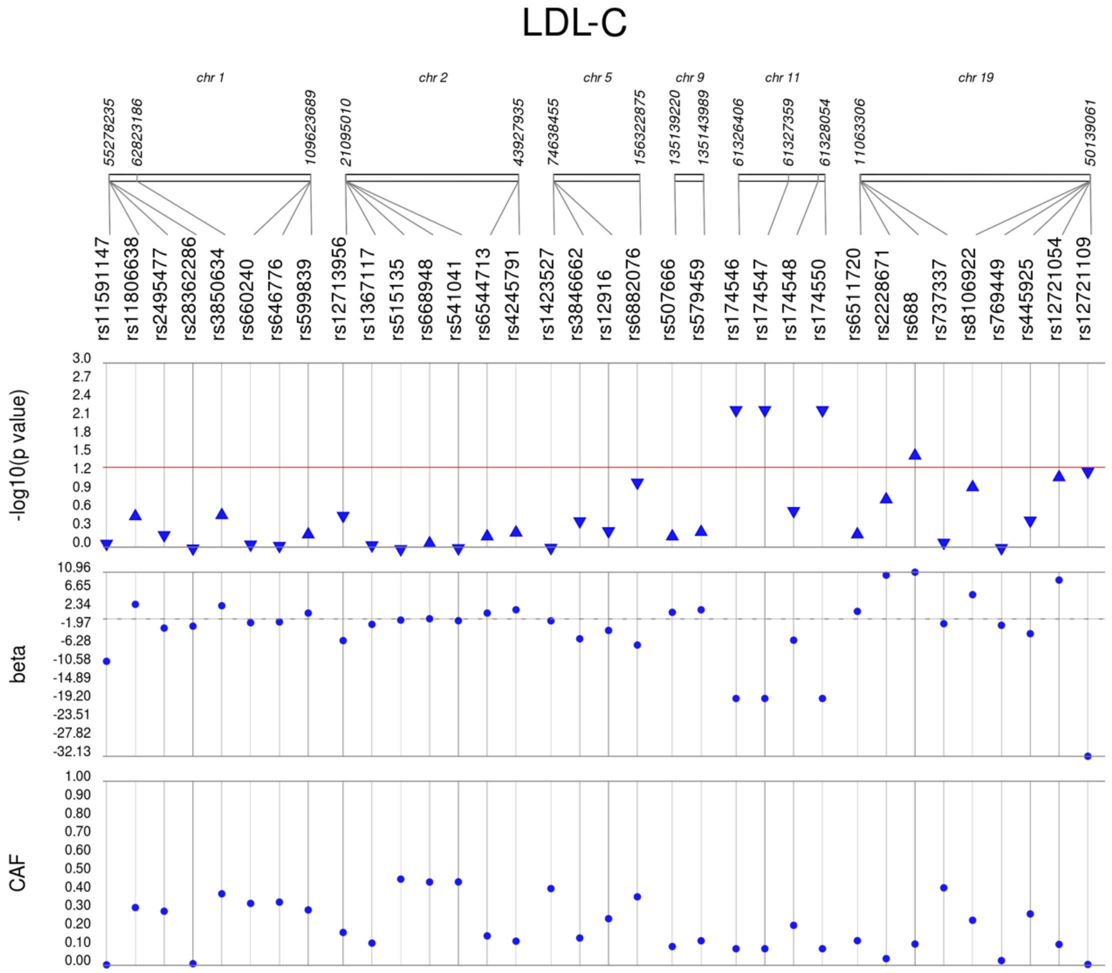
LDL-C
chr 1
chr 2
chr 5
chr 9
chr 11
chr 19
rs11591147
rs11806638
rs2495477
rs28362286
rs3850634
rs660240
rs646776
rs599839
rs12713956
rs1367117
rs515135
rs668948
rs541041
rs6544713
rs4245791
rs1423527
rs3846662
rs12916
rs6882076
rs507666
rs579459
rs174546
rs174547
rs174548
rs174550
rs6511720
rs2228671
rs688
rs737337
rs8106922
rs769449
rs445925
rs12721054
rs12721109
-log10(p value)
beta
CAF

C.

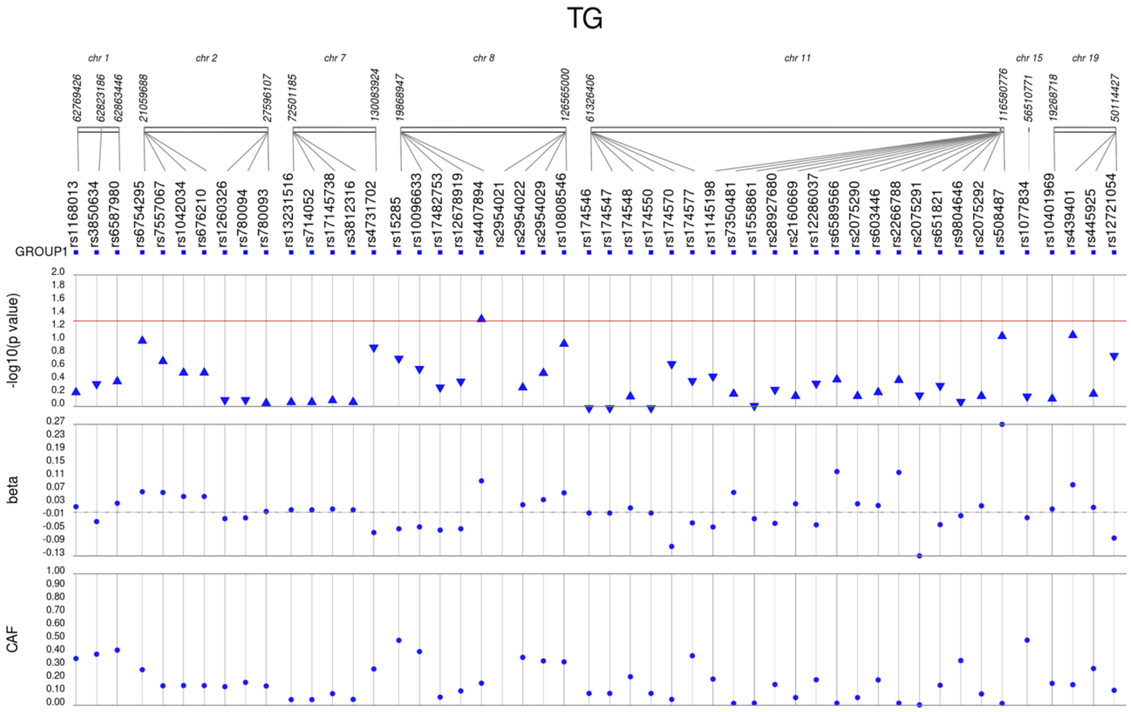
TG
chr 1
chr 2
chr 7
chr 8
chr 11
chr 15
chr 19
GROUP1
-log10(p value)
beta
CAF

D.

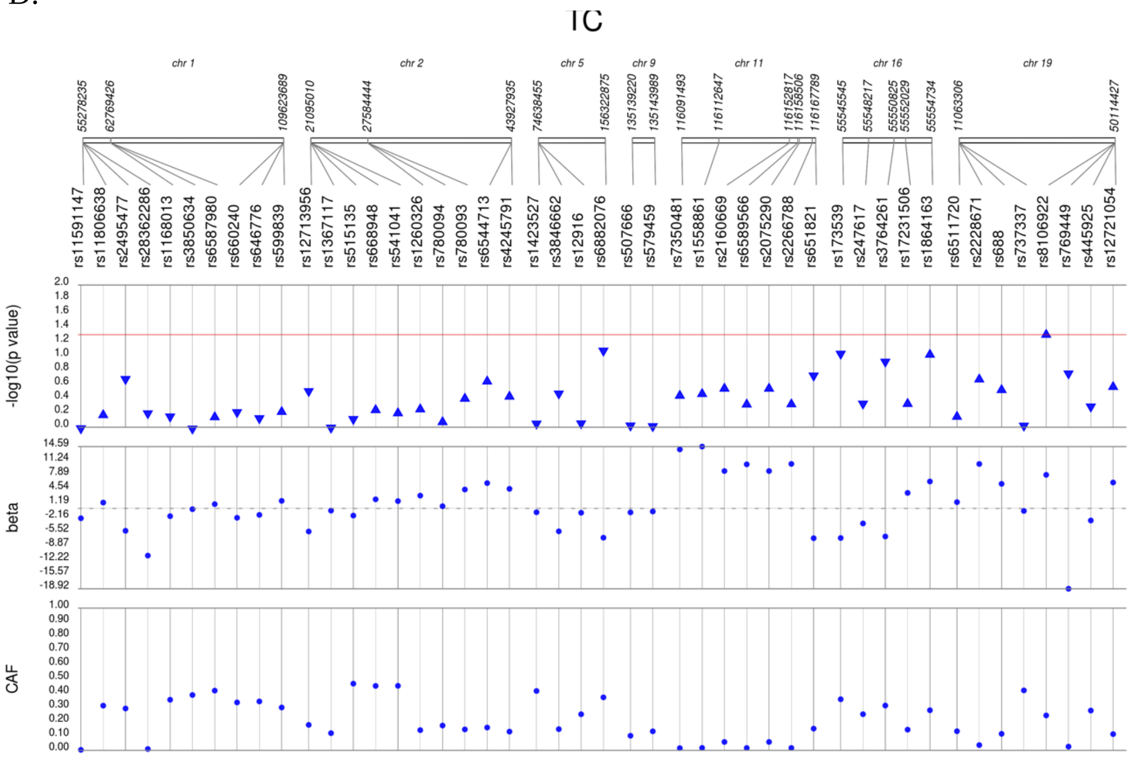
TC
chr 1
chr 2
chr 5
chr 9
chr 11
chr 16
chr 19
-log10(p value)
beta
CAF

C.

D.

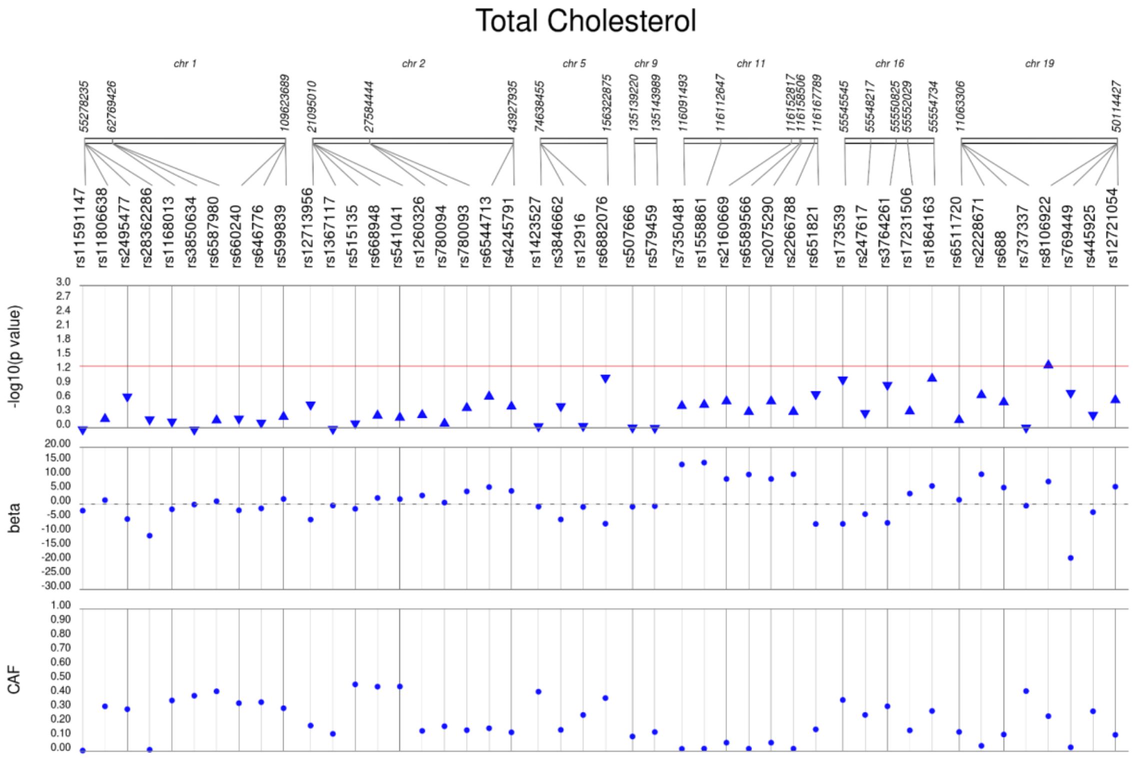
Total Cholesterol
chr 1
chr 2
chr 5
chr 9
chr 11
chr 16
chr 19
-log10(p value)
beta
CAF
