# Supplementary material for: Assessment of multi-population polygenic risk scores for lipid traits in African Americans
Source: PeerJ. 2023 May 16;11:e14910. doi: 10.7717/peerj.14910 (PMC10198155; doi:10.7717/peerj.14910)
Supplement: Supplemental Information 4 — We identified SNPs genotyped on the Metabochip that were reported as associated at p ≤ 5.0 × 10^−8^ with high density lipoprotein cholesterol (HDL-C), low density lipoprotein cholesterol (LDL-C), total cholesterol (TC), and triglycerides (TG) by (Hu et al., 2020). Shown per variant are chromosomal location, rs number, associated lipid trait, and genetic effect size. [file peerj-11-14910-s004.docx]

**Supplementary Table 1. SNPs associated with lipid traits used to calculate multi-population polygenic risk scores.** We identified SNPs genotyped on the Metabochip that were reported as associated at p≤5.0x10^-8^ with high density lipoprotein cholesterol (HDL-C), low density lipoprotein cholesterol (LDL-C), total cholesterol (TC), and triglycerides (TG) by Hu et al., 2020. Shown per variant are chromosomal location, rs number, associated lipid trait, and genetic effect size.

| **Chromosome** | **rs number** | **Associated lipid trait** | **Beta from Hu et al., 2020** |
| --- | --- | --- | --- |
| 2 | rs6754295 | HDL-C | 0.0487418128978 |
| 2 | rs7557067 | HDL-C | 0.0638536759132 |
| 2 | rs1042034 | HDL-C | -0.0659093997588 |
| 2 | rs676210 | HDL-C | 0.066099841798 |
| 7 | rs4731702 | HDL-C | 0.0469244975644 |
| 8 | rs9987289 | HDL-C | 0.0898413567202 |
| 8 | rs2126259 | HDL-C | 0.0846555461597 |
| 8 | rs15285 | HDL-C | 0.110260863471 |
| 8 | rs10096633 | HDL-C | 0.122332217472 |
| 8 | rs17482753 | HDL-C | 0.140889880499 |
| 8 | rs12678919 | HDL-C | 0.135269273934 |
| 8 | rs4407894 | HDL-C | 0.0702385857879 |
| 9 | rs2515629 | HDL-C | -0.0622166967375 |
| 9 | rs1883025 | HDL-C | -0.0569273894474 |
| 9 | rs1800978 | HDL-C | -0.0726214901544 |
| 11 | rs7350481 | HDL-C | 0.0938521762243 |
| 11 | rs1558861 | HDL-C | 0.0823836328501 |
| 11 | rs2160669 | HDL-C | 0.0902306588939 |
| 11 | rs6589566 | HDL-C | 0.0868624484373 |
| 11 | rs2075290 | HDL-C | 0.0889510667538 |
| 11 | rs2266788 | HDL-C | 0.0856909301923 |
| 15 | rs10468017 | HDL-C | 0.0731367164745 |
| 15 | rs2043085 | HDL-C | -0.0798767491117 |
| 15 | rs1532085 | HDL-C | -0.0803373828344 |
| 15 | rs1077834 | HDL-C | 0.0820328788846 |
| 15 | rs8034802 | HDL-C | 0.0582940519185 |
| 16 | rs9989419 | HDL-C | 0.0622135017508 |
| 16 | rs173539 | HDL-C | 0.176333325622 |
| 16 | rs247617 | HDL-C | 0.242878784706 |
| 16 | rs3764261 | HDL-C | 0.211507766764 |
| 16 | rs17231506 | HDL-C | 0.213376417197 |
| 16 | rs1800775 | HDL-C | 0.152202841916 |
| 16 | rs1864163 | HDL-C | -0.164352542546 |
| 16 | rs11076174 | HDL-C | -0.147343892378 |
| 16 | rs7205804 | HDL-C | 0.153821102638 |
| 16 | rs1532624 | HDL-C | 0.158562393321 |
| 16 | rs7499892 | HDL-C | -0.177884283256 |
| 16 | rs5883 | HDL-C | 0.182901731913 |
| 16 | rs5880 | HDL-C | -0.206798217578 |
| 16 | rs16942887 | HDL-C | 0.0804646961055 |
| 16 | rs255052 | HDL-C | 0.073490179233 |
| 18 | rs9958734 | HDL-C | 0.0813460262181 |
| 19 | rs737337 | HDL-C | -0.0822083947798 |
| 20 | rs1800961 | HDL-C | -0.154702502207 |
| 1 | rs11591147 | LDL-C | -0.548287339124 |
| 1 | rs11806638 | LDL-C | -0.0915943633283 |
| 1 | rs2495477 | LDL-C | -0.05883172493 |
| 1 | rs28362263 | LDL-C | -0.249481282475 |
| 1 | rs28362286 | LDL-C | -1.07142244262 |
| 1 | rs3850634 | LDL-C | -0.0516407299693 |
| 1 | rs660240 | LDL-C | 0.159535573853 |
| 1 | rs646776 | LDL-C | 0.160608877603 |
| 1 | rs599839 | LDL-C | 0.113740048718 |
| 2 | rs12713956 | LDL-C | -0.0856934736141 |
| 2 | rs1367117 | LDL-C | 0.105380333902 |
| 2 | rs515135 | LDL-C | 0.0769282913243 |
| 2 | rs668948 | LDL-C | 0.112721232049 |
| 2 | rs541041 | LDL-C | 0.112998152826 |
| 2 | rs4299376 | LDL-C | -0.0836493160971 |
| 2 | rs6544713 | LDL-C | -0.0795586819216 |
| 2 | rs4245791 | LDL-C | -0.0890923410319 |
| 5 | rs1423527 | LDL-C | 0.0717265335534 |
| 5 | rs3846662 | LDL-C | 0.0602637055649 |
| 5 | rs12916 | LDL-C | 0.0780453784262 |
| 5 | rs6882076 | LDL-C | 0.0474175479413 |
| 9 | rs507666 | LDL-C | 0.0856562399259 |
| 9 | rs579459 | LDL-C | 0.0676180853135 |
| 11 | rs174546 | LDL-C | -0.0659707558316 |
| 11 | rs174547 | LDL-C | -0.0661964516173 |
| 11 | rs174548 | LDL-C | -0.0547211104591 |
| 11 | rs174550 | LDL-C | -0.0661089868548 |
| 19 | rs6511720 | LDL-C | -0.185460720705 |
| 19 | rs2228671 | LDL-C | -0.117899696465 |
| 19 | rs688 | LDL-C | 0.0511617635514 |
| 19 | rs737337 | LDL-C | -0.0560302275683 |
| 19 | rs8106922 | LDL-C | 0.0530039518836 |
| 19 | rs769449 | LDL-C | 0.155096074545 |
| 19 | rs445925 | LDL-C | -0.166899867294 |
| 19 | rs12721054 | LDL-C | -0.171821977384 |
| 19 | rs12721109 | LDL-C | -0.388723272963 |
| 1 | rs11591147 | TC | -0.479929368523 |
| 1 | rs11806638 | TC | -0.0740508639157 |
| 1 | rs2495477 | TC | -0.0534084863132 |
| 1 | rs28362263 | TC | -0.218032893359 |
| 1 | rs28362286 | TC | -0.970092528551 |
| 1 | rs1168013 | TC | 0.0642731795269 |
| 1 | rs3850634 | TC | -0.0763079152302 |
| 1 | rs6587980 | TC | -0.0573999052834 |
| 1 | rs660240 | TC | 0.14035271251 |
| 1 | rs646776 | TC | 0.141449264652 |
| 1 | rs599839 | TC | 0.0991288214568 |
| 2 | rs12713956 | TC | -0.07888928067 |
| 2 | rs1367117 | TC | 0.0938111080038 |
| 2 | rs515135 | TC | 0.0628996898243 |
| 2 | rs668948 | TC | 0.098597183854 |
| 2 | rs541041 | TC | 0.0988949535018 |
| 2 | rs1260326 | TC | -0.0871652186064 |
| 2 | rs780094 | TC | -0.0803717138049 |
| 2 | rs780093 | TC | -0.0846935219817 |
| 2 | rs4299376 | TC | -0.0788109166572 |
| 2 | rs6544713 | TC | -0.0737557917852 |
| 2 | rs4245791 | TC | -0.0817188403756 |
| 5 | rs1423527 | TC | 0.068960875332 |
| 5 | rs3846662 | TC | 0.0634729689347 |
| 5 | rs12916 | TC | 0.0831456230472 |
| 5 | rs6882076 | TC | 0.0546362194052 |
| 9 | rs507666 | TC | 0.0830972264122 |
| 9 | rs579459 | TC | 0.0647650797521 |
| 11 | rs7350481 | TC | -0.0794658013349 |
| 11 | rs1558861 | TC | -0.0890519166545 |
| 11 | rs2160669 | TC | -0.0787929634324 |
| 11 | rs6589566 | TC | -0.0890383837312 |
| 11 | rs2075290 | TC | -0.0803654120417 |
| 11 | rs2266788 | TC | -0.0923106309713 |
| 11 | rs651821 | TC | -0.0693768597114 |
| 16 | rs173539 | TC | 0.0480420362019 |
| 16 | rs247617 | TC | 0.0574530622432 |
| 16 | rs3764261 | TC | 0.0537740565865 |
| 16 | rs17231506 | TC | 0.0576015390393 |
| 16 | rs1864163 | TC | -0.050841129829 |
| 19 | rs6511720 | TC | -0.161993579565 |
| 19 | rs2228671 | TC | -0.0977405520793 |
| 19 | rs688 | TC | 0.0506807975575 |
| 19 | rs737337 | TC | -0.071247236646 |
| 19 | rs8106922 | TC | 0.0465341920001 |
| 19 | rs769449 | TC | 0.141850197338 |
| 19 | rs445925 | TC | -0.109323555631 |
| 19 | rs12721054 | TC | -0.194181444826 |
| 1 | rs1168013 | TG | 0.0700332341976 |
| 1 | rs3850634 | TG | -0.082569974965 |
| 1 | rs6587980 | TG | -0.0620707584019 |
| 2 | rs6754295 | TG | -0.0490622057637 |
| 2 | rs7557067 | TG | -0.054668421223 |
| 2 | rs1042034 | TG | 0.0533307076279 |
| 2 | rs676210 | TG | -0.0545695488825 |
| 2 | rs1260326 | TG | -0.148277662531 |
| 2 | rs780094 | TG | -0.128346725389 |
| 2 | rs780093 | TG | -0.134301694501 |
| 7 | rs2240466 | TG | -0.128277794653 |
| 7 | rs13231516 | TG | -0.129507253285 |
| 7 | rs714052 | TG | -0.129627827868 |
| 7 | rs17145738 | TG | -0.112502945309 |
| 7 | rs3812316 | TG | -0.150242671454 |
| 7 | rs4731702 | TG | -0.0508343978538 |
| 8 | rs15285 | TG | -0.1113742316 |
| 8 | rs10096633 | TG | -0.139814182293 |
| 8 | rs17482753 | TG | -0.174304544658 |
| 8 | rs12678919 | TG | -0.14919674559 |
| 8 | rs4407894 | TG | -0.0682992427846 |
| 8 | rs2954021 | TG | -0.0680382836704 |
| 8 | rs2954022 | TG | -0.0536468783502 |
| 8 | rs2954029 | TG | -0.0553384114256 |
| 8 | rs10808546 | TG | -0.0548910811032 |
| 11 | rs174546 | TG | 0.069715131759 |
| 11 | rs174547 | TG | 0.0698311727217 |
| 11 | rs174548 | TG | 0.0560288226299 |
| 11 | rs174550 | TG | 0.0697608227649 |
| 11 | rs174570 | TG | 0.0645097868719 |
| 11 | rs174577 | TG | 0.0488567736398 |
| 11 | rs1145198 | TG | -0.059202691781 |
| 11 | rs7350481 | TG | -0.24988486014 |
| 11 | rs1558861 | TG | -0.24996488839 |
| 11 | rs28927680 | TG | 0.120350523403 |
| 11 | rs2160669 | TG | -0.222392987962 |
| 11 | rs12286037 | TG | 0.124904308238 |
| 11 | rs6589566 | TG | -0.253680879946 |
| 11 | rs2075290 | TG | -0.220879153082 |
| 11 | rs603446 | TG | -0.0719627429046 |
| 11 | rs2266788 | TG | -0.254151390865 |
| 11 | rs2075291 | TG | 0.381127445212 |
| 11 | rs651821 | TG | -0.17675167451 |
| 11 | rs9804646 | TG | -0.0766025285421 |
| 11 | rs2075292 | TG | -0.0919731958001 |
| 11 | rs508487 | TG | 0.115169049011 |
| 15 | rs1077834 | TG | 0.0481347650747 |
| 19 | rs10401969 | TG | -0.0692192325676 |
| 19 | rs439401 | TG | 0.0566284628756 |
| 19 | rs445925 | TG | 0.0632570096635 |
| 19 | rs12721054 | TG | -0.240939239262 |
